# Engineering linear, branched‐chain triterpene metabolism in monocots

**DOI:** 10.1111/pbi.12983

**Published:** 2018-10-16

**Authors:** Chase Kempinski, Zuodong Jiang, Garrett Zinck, Shirley J. Sato, Zhengxiang Ge, Thomas E. Clemente, Joe Chappell

**Affiliations:** ^1^ Plant Biology Program University of Kentucky Lexington KY USA; ^2^ Department of Pharmaceutical Sciences University of Kentucky Lexington KY USA; ^3^ Center for Biotechnology University of Nebraska‐Lincoln Lincoln NE USA; ^4^ Present address: Department of Soil and Crop Sciences Texas A&M University College Station TX 77843 USA

**Keywords:** triterpene, metabolic engineering, monocot, botryococcene, *Brachypodium*, *Sorghum*

## Abstract

Triterpenes are thirty‐carbon compounds derived from the universal five‐carbon prenyl precursors isopentenyl diphosphate (IPP) and dimethylallyl diphosphate (DMAPP). Normally, triterpenes are synthesized via the mevalonate (MVA) pathway operating in the cytoplasm of eukaryotes where DMAPP is condensed with two IPPs to yield farnesyl diphosphate (FPP), catalyzed by FPP synthase (FPS). Squalene synthase (SQS) condenses two molecules of FPP to generate the symmetrical product squalene, the first committed precursor to sterols and most other triterpenes. In the green algae *Botryococcus braunii*, two FPP molecules can also be condensed in an asymmetric manner yielding the more highly branched triterpene, botryococcene. Botryococcene is an attractive molecule because of its potential as a biofuel and petrochemical feedstock. Because *B. braunii,* the only native host for botryococcene biosynthesis, is difficult to grow, there have been efforts to move botryococcene biosynthesis into organisms more amenable to large‐scale production. Here, we report the genetic engineering of the model monocot, *Brachypodium distachyon*, for botryococcene biosynthesis and accumulation. A subcellular targeting strategy was used, directing the enzymes (botryococcene synthase [BS] and FPS) to either the cytosol or the plastid. High titres of botryococcene (>1 mg/g FW in T_0_ mature plants) were obtained using the cytosolic‐targeting strategy. Plastid‐targeted BS + FPS lines accumulated botryococcene (albeit in lesser amounts than the cytosolic BS + FPS lines), but they showed a detrimental phenotype dependent on plastid‐targeted FPS, and could not proliferate and survive to set seed under phototrophic conditions. These results highlight intriguing differences in isoprenoid metabolism between dicots and monocots.

## Introduction

**Figure 1:**
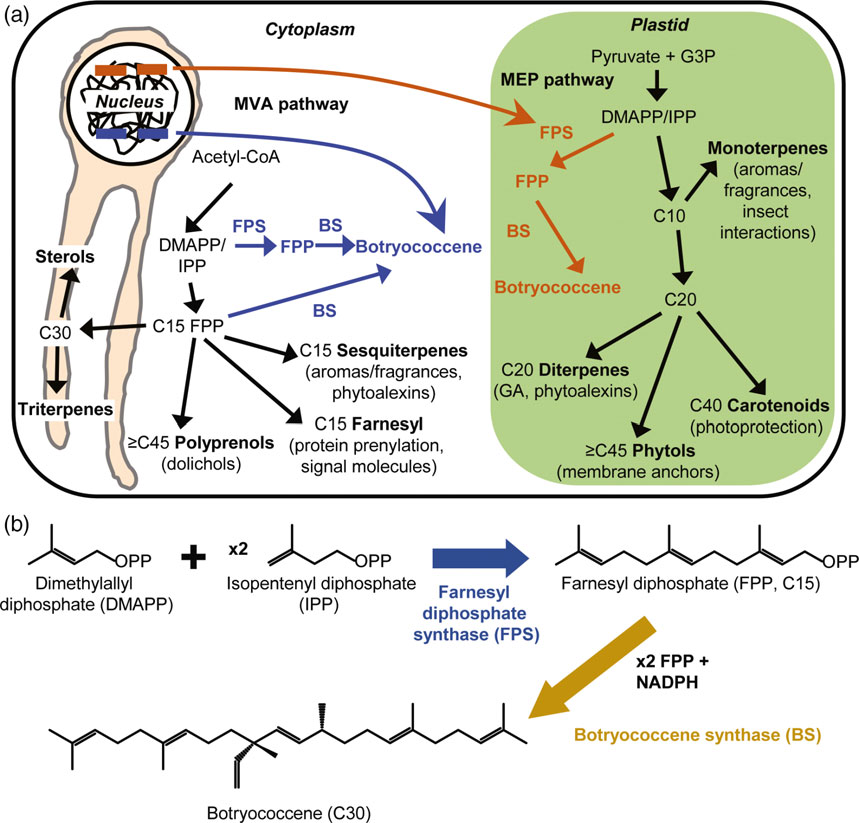
Schematic of the triterpene engineering strategy adapted from Wu *et al*. (2012). (a) Genes encoding for FPS, BS or both targeted to the cytoplasm (blue) or plastid (red) compartments were stably integrated into the nuclear genome of *B. distachyon*. The cytosol‐targeted enzymes utilize IPP/DMAPP from the MVA pathway to produce FPP by FPS, or can utilize FPP made natively by the endogenous FPS (lower blue arrow). The plastid‐targeted enzymes utilize IPP/DMAPP derived from the MEP pathway, but botryococcene production should require FPS because no endogenous mechanism(s) for FPP biosynthesis in the plastid compartment is known. (b) Summary of botryococcene biosynthesis via polymerization of DMAPP and two IPP molecules into FPP by FPS, followed by condensation of two FPP molecules into botryococcene catalyzed in a NADPH‐dependent manner by BS.

Isoprenoids are an incredibly diverse class of natural products, with tens of thousands currently discovered (Thulasiram *et al*., 2007; Tholl, 2015) and more being uncovered every year. These molecules have incredible complexity: from the carbon skeletons which arise via terpene synthase enzymes acting on diverse prenyl diphosphate substrates—which are all built from the condensation of five‐carbon precursors (IPP and DMAPP)—to the innumerable modifications/decorations which can occur, most often by cytochrome P450 enzymes (isoprenoid biosynthesis has been the subject of extensive review: Chappell, 2002; Kirby and Keasling, 2009; Hemmerlin *et al*., 2012). Important aspects are summarized briefly in Figure 1a. In plants, isoprenoid biosynthesis can proceed via two (mostly) independent pathways: the MVA pathway which occurs in the cytosol in association with the endoplasmic reticulum, or the methylerythritol phosphate (MEP) pathway which occurs within plastids. The primary substrate for the MVA pathway is acetyl‐CoA, and glyceraldehyde‐3‐phosphate and pyruvate for the MEP pathway, with both pathways yielding IPP and DMAPP. The condensation of IPP and DMAPP in a linear fashion gives rise to prenyl chains that vary in length by the number of prenyl units condensed together. The MVA pathway specializes in producing fifteen‐carbon prenyl chains in the form of FPP (Figure 1b), which gives rise to sesquiterpenes, and in eukaryotes, is condensed in a head‐to‐head manner by SQS to yield a thirty‐carbon triterpene, squalene, which is the first committed precursor to sterols. Conversely, in plants, the MEP pathway produces geranyl diphosphate (GPP), a ten‐carbon molecule derived from two prenyl units, and geranylgeranyl diphosphate (GGPP), a twenty‐carbon compound. GPP and GGPP give rise to the mono‐ and diterpenes, respectively. Head‐to‐head condensation of GGPP by phytoene synthase gives rise to the tetraterpene, phytoene, the precursor to carotenoids.

Because of their chemical diversity and complexity, isoprenoids have many uses as flavours and fragrances (e.g. menthol), in agriculture (e.g. gibberellins), in medicine (e.g. artemisinin) and in industry (e.g. polyisoprene or natural rubber). While these compounds are useful and valuable, they are often produced in small amounts by their natural hosts. With recent advances in biotechnology, we can now identify the genes encoding the enzymes involved in isoprenoid biosynthesis and transform these genetic blueprints into heterologous host species amendable to increased production and yields of desired products. Plants are an attractive platform for production of these compounds because, compared with microbes, they require very little infrastructure, are inexpensive to grow and utilize atmospheric CO_2_ as their carbon source. Much work has been done engineering isoprenoid metabolism in dicot species such as: tobacco, *Arabidopsis*, potato, mint, tomato, orange, *Camelina*, soybean and *Brassica napus* (for review see: Dudareva and Pichersky, 2008; Lange and Ahkami, 2013; Kempinski *et al*., 2015; Arendt *et al*., 2016; Tatsis and O'Connor, 2016). Conversely, very little isoprenoid metabolic engineering has been done in monocot species, where their recalcitrance to *Agrobacterium*‐mediated transformation has hindered these efforts (Cheng *et al*., 2004; Sood *et al*., 2011). However, there have been some important results, such as the generation of ‘golden rice’ (Ye *et al*., 2000), tocotrienol and tocopherol engineering in *Zea mays* (Cahoon *et al*., 2003), carotenoid engineering in *Z. mays* (Zhu *et al*., 2008) and rice (Breitenbach *et al*., 2014), sesquiterpene engineering for defense in *Z. mays* (Robert *et al*., 2013) and triterpene engineering in rice (Huang *et al*., 2015a,2015bB; Inagaki *et al*., 2011).

Engineering of triterpenes is still a relatively new proposition. However, these compounds have great interest due to their many uses. Focus has primarily been on cyclized triterpenes arising by the action of an oxidosqualene synthase (OSC) on 2,3‐oxidosqualene, which in turn, is generated from squalene by squalene epoxidase (SQE). The formation of 2,3‐oxidosqualene is common to all eukaryotes as it is cyclized to lanosterol in animals and fungi, being the precursor to cholesterol and ergosterol, respectively. In plants, 2,3‐oxidosqualene is primarily cyclized to cycloartenol, the precursor to the phytosterols (Nes, 2011). While sterols and their derivatives are interesting due to their potential uses as anti‐cholestemic agents in humans and for the nutritional benefits of phytosterols (Amir Shaghaghi *et al*., 2013), the complex structures and modifications that occur on cyclized triterpenes through the action of OSCs to generate non‐sterol carbon backbones have also triggered much intrigue due to their medicinal potential (for review see: Moses *et al*., 2013). However, squalene is not the only linear triterpene possible and different condensations of FPP (rather than the 1‐1′ linkage that results in squalene) can generate unique thirty‐carbon compounds with a similar, but striking difference to squalene. Of particular interest is botryococcene, which is only naturally produced by the green algae, *Botryococcus braunii* (Bell *et al*., 2014; Niehaus *et al*., 2011). *B. braunii* has three well‐characterized races, each of which specializes in producing large amounts of certain chemical types. Race A produces alkadienes and trienes (which arise primarily from fatty acid metabolism), race L produces the tetraterpenoid, lycopadiene, and race B produces the triterpenoids, botryococcene and methylated derivatives of botryococcene and squalene (Metzger and Largeau, 2005).

*B. braunii* is an evolutionarily ancient organism and its hydrocarbons, botryococcene/squalene and the methylated derivatives, are believed to contribute to considerable amounts of the petroleum deposits found throughout the world (Derenne *et al*., 1997; Glikson *et al*., 1989). Indeed, methylated botryococcene (and squalene) can be cracked into usable petroleum feedstocks, which could serve as drop‐in replacements for fossil fuels obtained from geological deposits (Hillen *et al*., 1982; Kimura *et al*., 2012; Niehaus *et al*., 2011; Tracy *et al*., 2011). However, *B. braunii* itself is generally not an attractive source for producing these oils due to its very slow growth when compared with algae‐like *Chlamydomonas reinhardtii*, although there have been attempts to optimize its culture (Casadevall *et al*., 1985; Frenz *et al*., 1989; Khatri *et al*., 2014; Yoshimura *et al*., 2013). As an alternative to this, the mechanisms that *B. braunii* uses to biosynthesize botryococcene have been investigated and characterized (Niehaus *et al*., 2011; Okada *et al*., 2004). Botryococcene biosynthesis proceeds through two enzymes: squalene synthase‐like (SSL)‐1 and SSL‐3. SSL‐1 catalyzes the first condensation of two molecules of FPP into the presqualene diphosphate intermediate, which is then catalytically reduced by SSL‐3 to produce the 1‐3′ linkage of the two FPP molecules, yielding botryococcene (Figure 1b; Niehaus *et al*., 2011). A catalytic fusion of SSL‐1 and SSL‐3 (hereafter referred to as BS—botryococcene synthase [Figure 1b]) was seen to have elevated botryococcene accumulation in vivo using a yeast system, and this catalytic fusion has been transformed into a variety of heterologous organisms (i.e. *Escherichia coli*,* Rhodobacter capsulatus*,* Nicotiana tabacum* and *Saccharomyces cerevisiae*) that may be more suitable for production than algae (Bell *et al*., 2014; Jiang *et al*., 2016; Khan *et al*., 2015; Zhuang and Chappell, 2015).

In this study, we investigated the potential for monocots (and especially grasses) to produce heterologous triterpenes, specifically, botryococcene. We tested this using the model monocot, *Brachypodium distachyon,* then extended these observations to *Sorghum bicolor*. *B. distachyon* has a published genome and many other attributes which make it a model species for working with monocots (Draper *et al*., 2001; Mur *et al*., 2011; Vogel and Bragg, 2009). An important attribute which was critical to this study is an accessible *Agrobacterium*‐mediated transformation protocol (Alves *et al*., 2009; Vogel *et al*., 2006). While *B. distachyon* is an annual species, agronomic and perennial grasses have the potential to make excellent production platforms due to their ability to produce multiple harvests from one planting. Thus, we wanted to demonstrate the ability to use other members of the Poaceae‐like *Sorghum* as production platforms for hydrocarbon isoprenoids (i.e. botryococcene). To accomplish this, we used strategies previously pioneered in dicot species: subcellular targeting of heterologous FPS and BS to utilize prenyl precursors from the two isoprenoid pathways present in plants. BS, with or without FPS, was targeted to the cytosol to evaluate carbon derived from the MVA pathway, or the plastid to evaluate carbon derived from the MEP pathway (Augustin *et al*., 2015; Jiang *et al*., 2016; Wu *et al*., 2006, 2012). The results of these engineering efforts produced unexpected observations. Contrary to results with the majority of dicot engineering, we were able to obtain high amounts of botryococcene using a cytosol‐directed strategy. In addition, our plastid‐targeting strategy resulted in an aberrant phenotype, which prohibited the plants from thriving under phototrophic conditions. However, this phenotype was observed only in plants engineered with plastid‐targeted FPS, plastid‐targeted BS alone did not induce it. These observations are intriguing in that they show the ability to produce a non‐native hydrocarbon in high amounts in the cytosol of a grass species, and the precarious nature of engineering isoprenoid metabolism in the plastid of *B. distachyon*. We also investigated if these results translated to a monocot crop species, *S. bicolor*. These findings underscore that paradigms developed from one species or phylogeny may not apply across the plant kingdom and there are differences that remain to be explored and exploited.

## Results

### Elevated levels of cytosolic botryococcene increased by including an FPS in combination with BS

**Figure 2:**
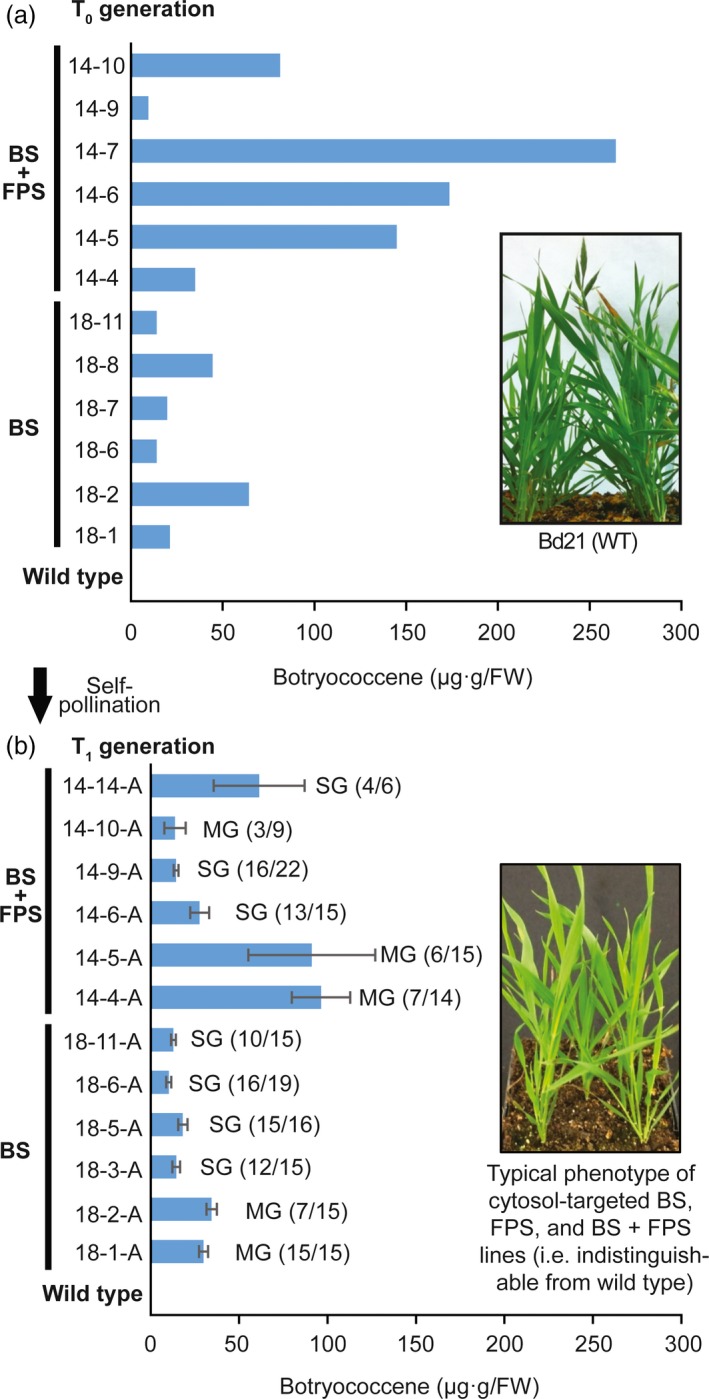
Botryococcene accumulation in *B. distachyon* engineered with genes targeting BS ± FPS to the cytoplasm. (a) Botryococcene levels in leaves (second and third leaves from a three‐leaf culm) of primary transformants (T_0_ generation) were determined by GC‐MS. The T_0_ plants were allowed to self‐pollinate and 6‐22 seeds of each T_1_ generation were germinated and grown for up to 4 months. (b) Botryococcene accumulation in leaves of ~4‐month‐old plants of individual T_1_ generation plants was subsequently determined, with the average ± standard error shown for only those accumulating botryococcene. Primary transformants are identified by the transformation event (e.g. 14 or 18) and the resulting transgenic plant (e.g. 14‐4). The subsequent T_1_ progeny are identified by their transformation event, the primary transformant identifier, and the individual T_1_ plant (e.g. 14‐4‐1) or T_1_ population examined (e.g. 14‐4‐A). The bottom inset picture depicts the overall phenotype of a T_1_ generation relative to wild type plants (top inset picture). Notation to the right of the histobars in the lower panel indicate those T_1_ populations which demonstrated segregation of a single gene (SG) phenotype (3:1), while “MG” indicates those populations where the transgenes did not segregate as a simple, single genetic insertion (presence of the transgene was based on botryococcene accumulation and Χ^2^ test, α = 0.05). For the T_1_ lines in the lower panel, the botryococcene level was measured in individual sibling plants and the average amongst those accumulating botryococcene presented. The number of individual T_1_ siblings accumulating botryococcene versus total number of siblings screened is noted as well (# positive/# screened).

Engineering BS activity (under the *Panicum virgatum UBIQUITIN2* promoter, 5′ UTR, and intron 1 [Mann *et al*., 2011]) into the cytosol of *B. distachyon* resulted in botryococcene accumulation in modest amounts (on the order of ~40 μg/g FW) in T_0_ plants which was consistent with levels seen in T_1_ botryococcene accumulating lines resulting from self‐pollination (Figure 2). Importantly, botryococcene could be increased by co‐expressing a heterologous FPS (under the constitutive *Z. mays UBIQUITIN 1* promoter, with 5′ UTR, and intron 1 [Christensen and Quail, 1996]) along with BS, both targeted to the cytosolic compartment. Some lines of younger T_0_ plants showed botryococcene levels >100 μg/g FW (Figure 2a), but the levels of botryococcene accumulation increased with developmental age of the tissue and with overall plant age in general (Figure S1). Some extractions showed accumulation >1 mg/g FW (in mature leaves of flowering and senescing plants of the high‐producing line, 14‐6). To the best of our knowledge, these are some of the highest levels of engineered triterpene accumulation in the cytosol of leaves of any higher plant, and specifically monocots. These botryococcene accumulating plants were phenotypically indistinguishable from wild type (compare phenotype of BS + FPS lines inset in Figure 2 bottom panel to wild type inset in top panel). The chemical integrity of botryococcene produced by *B. distachyon* was verified by GC‐MS and ^1^H‐NMR (Figure S2).

To check if the higher level of botryococcene accumulation in the BS + FPS lines was perhaps due to differing activity levels of BS, we examined BS activity in crude leaf lysates of lines engineered with BS and BS + FPS (Figure S3A). BS specific activity in crude leaf lysates did not appear to correlate with botryococcene accumulation when the BS‐only expressing lines (18‐1 and 18‐2) were compared to a lower‐ and higher‐accumulating T_0_ BS + FPS expressing lines, 14‐4 and 14‐7 respectively. Because the activity of BS was not that different among the four lines examined (although it did appear lower in the tissue examined from line 14‐4) we interpreted this as BS activity not being the limiting factor on botryococcene accumulation in the cytosol‐engineered lines, but more likely it was the availability of the precursor, FPP. Interestingly, in the tissue examined, 14‐4 accumulated lower amounts of botryococcene and displayed lower BS activity levels when compared with the higher accumulating 14‐7 line (Figure S3B). The lower BS activity in 14‐4 could explain its reduced ability to accumulate botryococcene when compared with other T_0_ BS + FPS lines. The increased activities in Figure S3B may reflect using older plant tissue for these determinations.

### Phytosterol levels in cytosol‐targeted botryococcene lines are comparable to wild type

**Figure 3:**
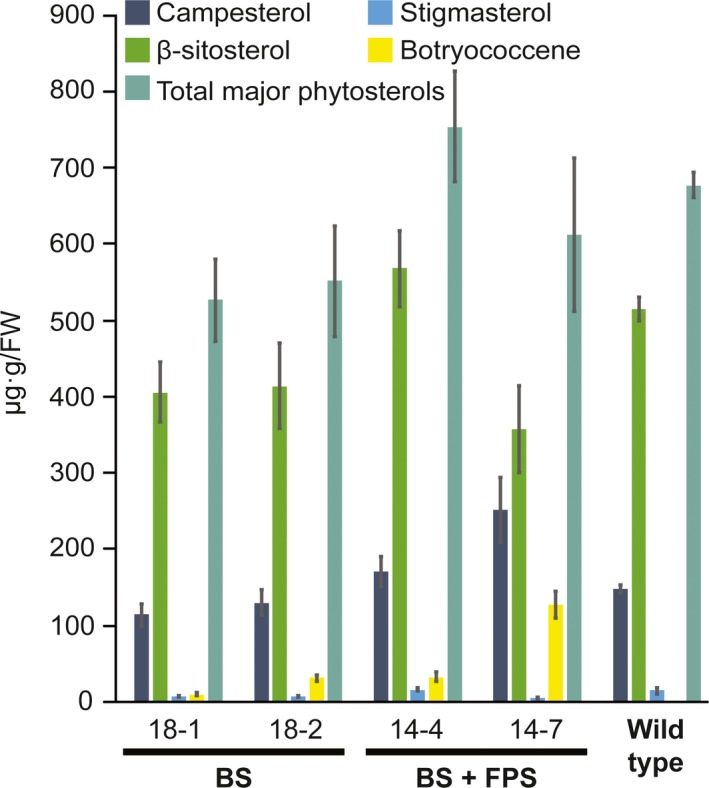
Comparison of the major phytosterol levels in the transgenic lines engineered for cytosolic BS and BS+FPS activity relative to control (wild type) non‐transgenic lines. Total sterols were extracted from leaves of ~3‐month‐old plants, derivatized, and quantified by GC‐MS. The botryococcene levels were determined from the same GC‐MS chromatograms. Assays were performed in triplicate with the average determinations reported ± standard errors.

Because cytosolic FPP is also the precursor to phytosterols, we examined the phytosterol contents in various BS and BS + FPS engineered lines. While the ratios of certain major phytosterols may be altered between some of the lines examined in comparison to wild‐type plants (Figure 3), the overall levels of phytosterols were not significantly different between the various transgenic lines examined and the wild type (α = 0.05, Student's *t*‐test, Figure 3). While BS‐only engineered lines 18‐1 and 18‐2 appear to have reduced total major phytosterol levels on average, these were not statistically significant.

We evaluated if there were any potential changes on transcript levels of MVA pathway genes due to the introduced metabolism in botryococcene‐accumulating T_2_ plants engineered with BS or BS+FPS (Figure S4). Genes encoding enzymes involved in important regulatory steps were analysed: acetoacetyl‐CoA thiolase (*AACT*), two potential HMGR isoforms (*HMGR1/HMGR2*), *SQS*, and two potential isoforms of isopentenyl diphosphate isomerase (*IDI1*/*IDI2*). Examining the average transcript levels in plants derived from three independent transformation events showed no significant differences compared with WT. However, both *SQS* and *AACT* transcripts were modestly (statistically significant) altered (higher and lower, respectively) in lines engineered with BS‐only compared with the WT (α = 0.05, Student's *t*‐test, Figure S4).

### Plastid‐targeted strategy of botryococcene biosynthesis results in accumulation but an aberrant phenotype due to FPS

**Figure 4:**
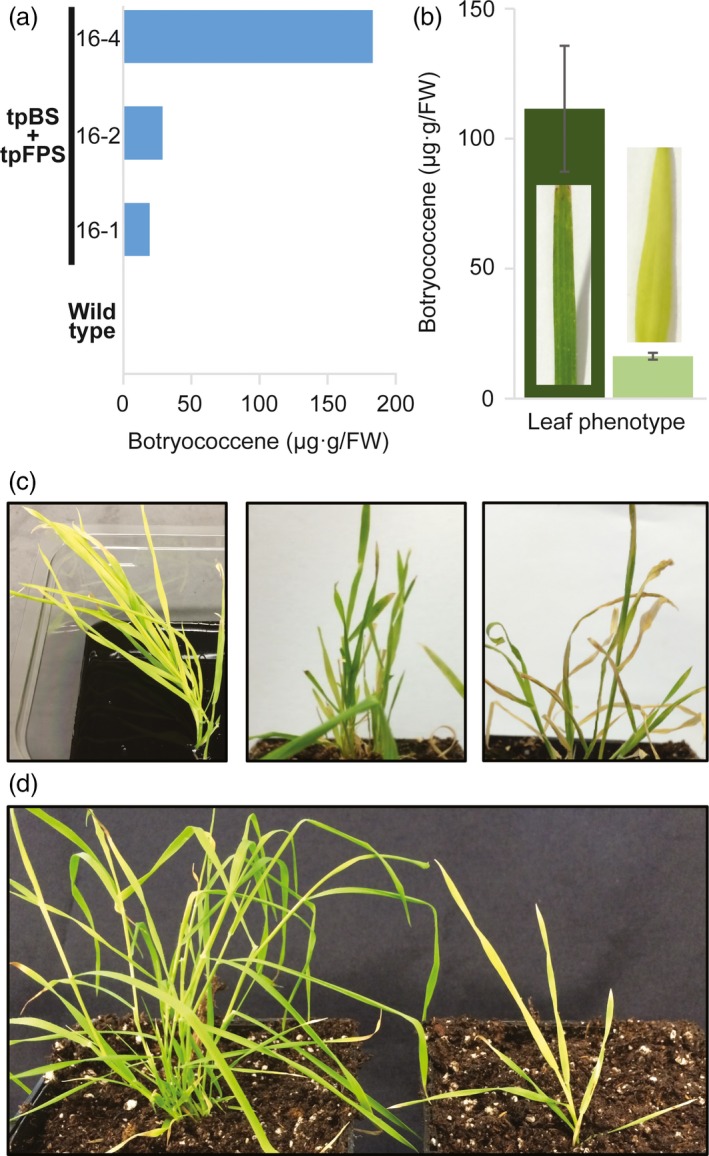
Plastid targeting of botryococcene metabolism has negative impacts on growth performance. (a) T_0_ lines with plastid‐targeted tpBS + tpFPS accumulate botryococcene during the plant transformation/regeneration protocol (botryococcene determinations were done with a mixture of leaves). (b) Some leaves would eventually green and botryococcene accumulation did increase with age/greening (leaves selected based on phenotype/age; *n *=* *3 ± standard errors). (c) However, tpBS + tpFPS plants exhibit a chlorosis (left panel, supplied with carbon) which was never overcome and ultimately lead to plant death when plants are moved to soil (middle image, plants moved to soil, and far right image, plants prematurely senescing after a few weeks under phototrophic conditions before setting seed—middle and right image of same plant). (d) Plastid‐targeted BS only lines did not exhibit the adverse growth effects (left plant, tpBS; right plant, tpBS + tpFPS) but also did not accumulate substantial levels of botryococcene.

Previous isoprenoid engineering efforts have shown the ability to obtain the highest yields of FPP‐derived products when a heterologous FPS and terpene synthase are targeted to the plastid of dicot plants (Augustin *et al*., 2015; Jiang *et al*., 2016; Wu *et al*., 2006, 2012). To this end, we sought to determine if high levels of botryococcene could be obtained using a plastid‐targeted strategy of FPS and BS in *B. distachyon*. We assumed that plastid‐targeted FPS would be necessary because the triterpene precursor, FPP, is not known to be produced endogenously within the plastids. Plastid‐targeted BS + FPS *B. distachyon* lines did accumulate botryococcene (Figure 4a), with regenerating lines accumulating ~20‐100 μg/g FW. These T_0_ lines also showed increasing accumulation with age. Some of the more mature leaves would eventually become dark green, but did not show robust lengthening typically associated with older leaves of wild type‐like plants. Darker green leaves showed a much higher level of botryococcene accumulation, approximately fivefold higher when compared with pale leaves (Figure 4b). However, even these more mature tissues could not match the higher levels obtained in very mature leaves of cytosolic lines grow on soil (Figure S1B).

Interestingly, and unlike studies done in dicots, constitutive expression of FPS in the plastids of *B. distachyon* caused a yellow, chlorotic phenotype in regenerating T_0_ plantlets (Figure 4c). These plants were unable to survive on soil under photosynthetic conditions. Growth stagnated followed by senescence on soil without producing seeds (see far right panel in Figure 4c). This phenotype did not seem to be caused by plastid‐targeted BS, as those regenerated plantlets were healthy, and have a wild‐type phenotype (Figure 4d, left plant). Thus, it seems that targeting a heterologous FPS to the plastid in the *B. distachyon* plastid caused a disruption in essential metabolic process(es), which prevented the plant from successfully producing enough carbohydrate from photosynthesis for survival. Plantlets engineered with only cytosolic‐targeted FPS did not show the aberrant phenotype.

To verify that BS expression was not abnormally different between the two engineering strategies we examined BS enzyme activity in two cytosolic‐targeted BS + FPS lines and a plastid‐targeted BS + FPS line. BS activity appeared approximately the same in the plastid‐targeted line examined (16‐4) when compared to a high botryococcene accumulating cytosolic line (14‐7) (Figure S3B). We interpret these results as reflecting the upper limits on BS activity using the *P. virgatum UBIQUITIN2* constitutive promoter complex, which is independent of subcellular targeting. This also supported the notion that it was not BS activity causing the inability of the tpBS + tpFPS plants to survive under phototrophic conditions.

### Cytosolic‐targeted botryococcene biosynthesis results in accumulation of an oxidobotryococcene

**Figure 5:**
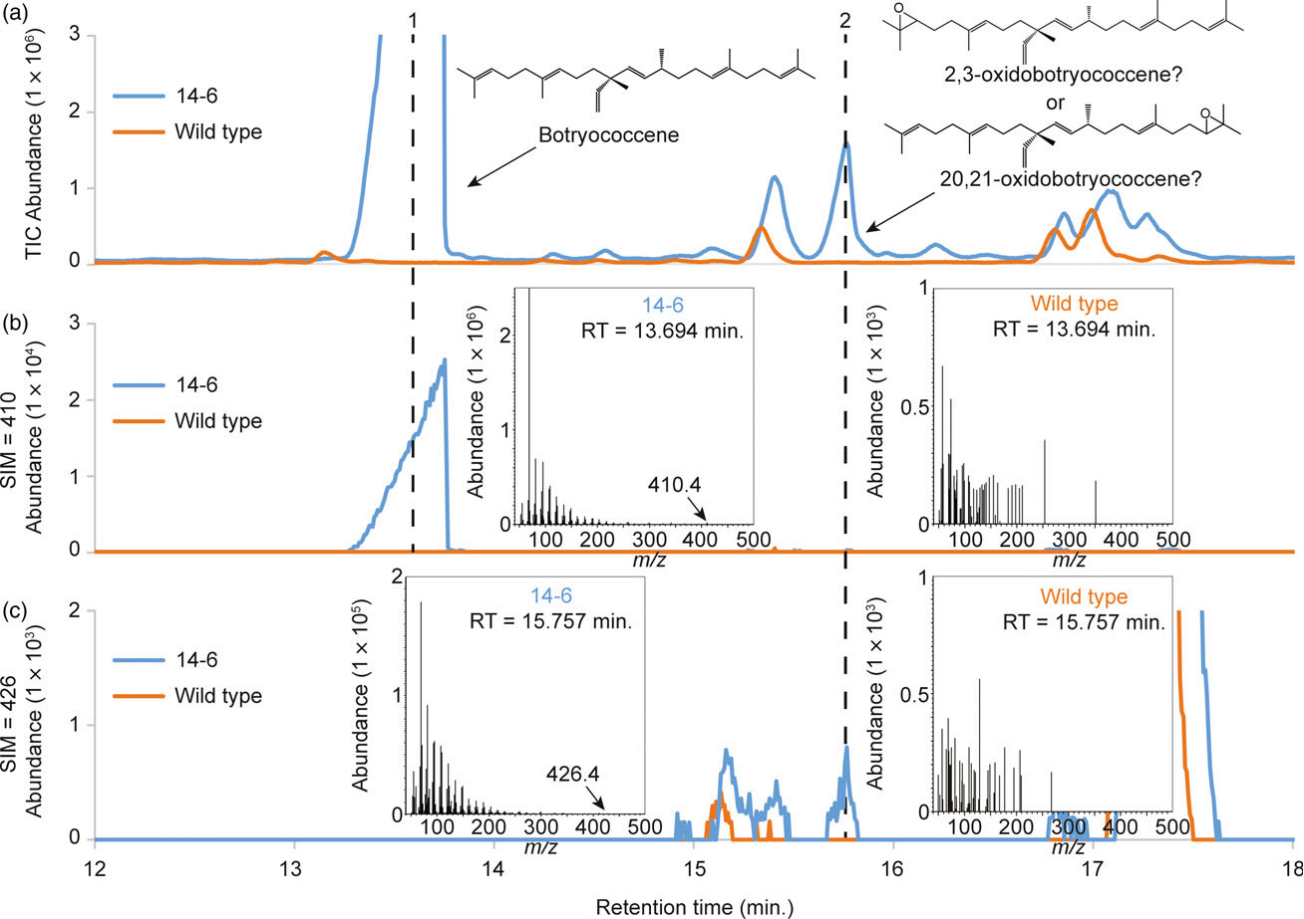
GC‐MS profiles of triterpenes accumulating in non‐transgenic control (wild type, orange trace) *B. distachyon* and a transgenic line engineered with BS + FPS targeted to the cytoplasm (blue trace). Extracts were prepared from leaves of comparable aged plants (~8 months old) and the botryococcene and putative oxidobotryococcene peaks annotated with chemical structures and MS profiles. Panel a provides chromatograms with total ion monitoring, while panels b and c use selective ion monitoring for ions of 410 and 426 *m/z*, diagnostic for botryococcene and oxidotriterpene, respectively.

Upon close examination of the GC‐MS chromatograms from simple hexane extracts from cytosol‐targeted BS + FPS lines, a new peak was observed in comparison to wild type and tpBS + tpFPS lines. This new peak eluted shortly after botryococcene (peak 1 in Figure 5a) and the concentration was proportional to botryococcene accumulation (peak 2 in Figure 5a). This new peak showed a mass spectrum similar to botryococcene (compare MS from Figure 5b,c) but had an apparent molecular parent ion of 426. Given the particular mass spectra of peak 2 in Figure 7c, and the apparent parent ion of 426, this peak was suspected to be an epoxidated form of botryococcene — oxidobotryococcene. This molecule is likely produced by the endogenous *B. distachyon* squalene epoxidase.

Because squalene is a symmetrical molecule, it can effectively be epoxidated on either end by SQE and maintain the same isomeric structure. However, because botryococcene is an asymmetrical molecule, depending on what terminal end of the molecule was acted upon by SQE, it could give rise to 2,3‐oxidobotryococcene (Figure S5A) or 20,21‐oxidobotryococcene (Figure S5B). If the endogenous SQE preferentially epoxidates one side of botryococcene over the other, one should be able to deduce that from the mass spectrum. Preferential epoxidation on either side should give rise to specific mass fragments which can only be produced from 2,3‐oxidobotryococcene or 20,21‐oxidobotryococcene (see the highlighted fragments in Figure S5A,B). However, it is important to note that since rearrangements following electron ionization are common, the highlighted fragments are not necessarily an exhaustive list of the possible indicator ions for a particular isoform. All of the ions shown in Figure S5A, B are found in the mass spectra for the putative oxidobotryococcene compound (Figure 5c). However, ion 205 was not found, but 203 was, which could represent a ‐2H+ loss for that particular fragment similar to what was reported by Oyugi *et al*. (2011). Since indicator ions for both compounds are found in the mass spectrum for the putative oxidobotryococcene peak, it could be that both isoforms are synthesized in planta and co‐elute under the current GC‐MS conditions.

**Figure 6:**
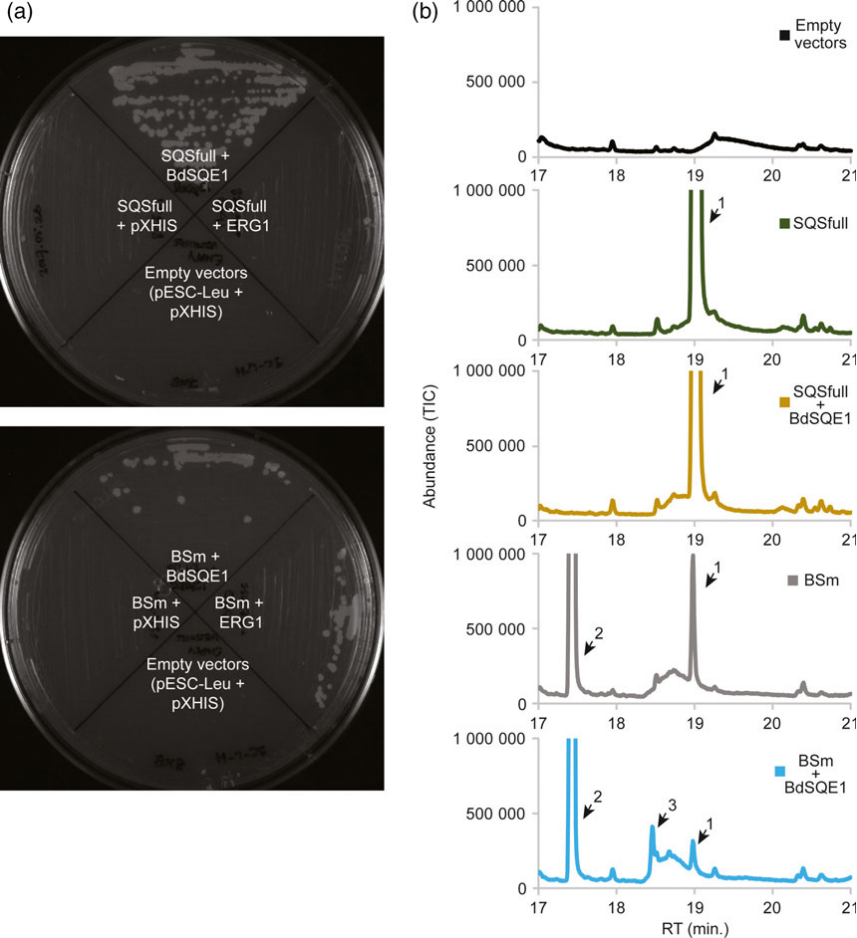
Functional characterization of *B. distachyon* squalene epoxidase 1 (BdSQE1). (a) Yeast line ZXB, which has deletion mutants for the native squalene synthase (∆*erg*9) and squalene epoxidase (∆*erg*1) genes, was transformed with two plasmid vectors without any inserted genes, or with one vector harbouring either the native yeast squalene synthase gene (*SQSfull*) (upper plate) or the botryococcene synthase gene (*BSm*) (lower plate) and the second vector harbouring either the *B. distachyon* squalene epoxidase 1 (*BdSQE1*) or the yeast native squalene epoxidase gene (*ERG*1) or no gene insert (Empty vector [*pXHIS*]). Single transformants selected for growth on plates containing exogenous sterol where subsequently streaked onto plates without exogenous sterol and scored for growth (restoration of sterol prototrophy) after 7 days. (b) Single colonies of the indicated transformants were also inoculated into liquid media containing exogenous sterol and the cultures grown to saturation. Cells were collected and hexane extracts profiled by GC‐MS. Peaks are numbered, 1: squalene, 2: botryococcene and 3: putative oxidobotryococcene. The MS for the new peak compound appearing in the BSm + BdSQE1 line at retention time 18.466 min (peak 3) matches the MS for the putative oxidobotryococcene found *in planta* (Figure 5 and Figure S6).

To confirm the identity of the putative oxidobotryococcene and its origin by the action of the native SQE, we cloned the endogenous *B. distachyon* SQE (*BdSQE1*) for heterologous expression studies. Only one putative gene was identified in the *B. distachyon* genome upon using the Arabidopsis *SQE1* gene (*AT1G58440*) as query in a tBLASTn search: *BRADI1G69170*. We expressed this gene in a yeast line engineered for high triterpene accumulation, ZXB, which has the endogenous *SQS* (*ERG9*), and *SQE* (*ERG1*) genes knocked out, as well as addition of a truncated HMGR to increase isoprenoid accumulation (Zhuang and Chappell, 2015). The ZXB line was transformed with one plasmid expression vector harbouring either the native yeast squalene synthase gene (*SQSfull*) or the botryococcene synthase (*BSm*) gene or neither gene (empty), plus a second expression vector harbouring either the native yeast squalene epoxidase gene (*ERG*1) or the putative *B. distachyon* squalene epoxidase gene (*BdSQE*1) or neither, and the initial transformants selected on plates containing exogenous sterol. Individual colonies from each transformation were streaked onto plates without exogenous sterol to determine if the various gene combinations restored sterol prototrophy (Figure 6a). As expected, transformants containing empty expression vectors (or with only *SQSfull*) did not grow (upper plate). Transformants with both the yeast squalene synthase and epoxidase genes also did not restore growth (right quandrant, upper plate). While this may seem counterintuitive, the ZXB line is engineered for enhanced biosynthesis of FPP, and when both the native yeast *SQSfull* and *ERG1* genes are over‐expressed the funnel of additional carbon into the ergosterol biosynthetic pathway is thought to lead to the buildup of toxic intermediates and shunt products (Linscott *et al*., 2016). However, sterol prototrophy was restored when *SQSfull* was co‐expressed with *BdSQE1* (upper quandrant, upper plate).

When the ZXB line was transformed with the botryococcene synthase gene (*BSm*), no growth was apparent without exogenous sterol (lower plate, Figure 6a). When the *BSm* gene was co‐expressed with *ERG1* or *BdSQE1*, sterol prototrophy was restored. BS can catalyze the biosynthesis of a small amount squalene (at less than 10% of catalysis for botryococcene), which can account for the modest growth observed on this plate.

Select colonies from the transformations were also grown in liquid cultures with exogenous sterol for subsequent chemical profiling (Figure 6b). When ZXB transformed with the yeast squalene synthase gene alone or in combination with the putative *B. distachyon* squalene epoxidase, abundant amounts of squalene accumulated. Only when ZXB was transformed with the *BSm* gene and with the *BdSQE1* gene did a new peak appear (peak 3, retention time ~18.5 min). The MS of this new peak matched that of the putative oxidobotryococcene produced *in planta* (Figure S6).

### The agronomically relevant monocot crop species, *Sorghum bicolor,* can also accumulate botryococcene in the cytosol

Because *B. distachyon* is a model laboratory species for monocot studies, we wished to evaluate if the results observed here translated to the agriculturally relevant monocot, *S. bicolor*. Transgenic *S. bicolor* lines were generated expressing cytosolic‐targeted BS only (two lines), BS + FPS (three lines) and plastid‐targeted tpBS + tpFPS (two lines). T_1_ transgenic plants were grown and screened for botryococcene accumulation after 5 weeks and analysed for the presence of the *BS* gene in the genome if they did not exhibit botryococcene accumulation. Interestingly, while multiple plants in both independent lines of tpBS + tpFPS indicated genomic presence of the *BS* gene, neither exhibited botryococcene accumulation. The lack of botryococcene production in *S. bicolor* tpBS + tpFPS lines is additional evidence the monocot plastid appears sensitive to perturbation when compared to efforts in dicots.

**Figure 7:**
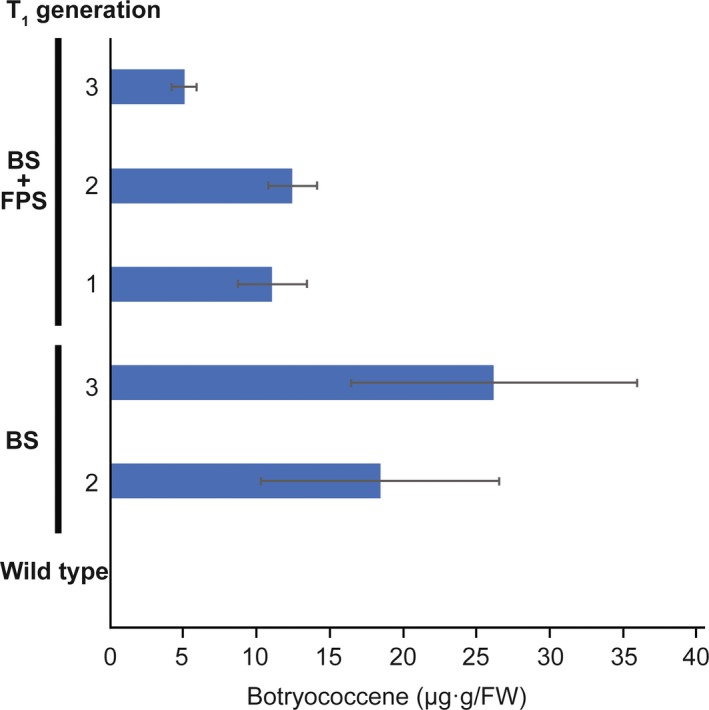
Botryococcene accumulation in *S. bicolor* engineered with genes targeting BS** ± **FPS to the cytoplasm. Botryococcene accumulation in the third through sixth leaves of ~4.5‐month‐old plants of individual T_1_ generation plants was subsequently determined, with the average** ± **standard error (SE) shown for only those accumulating botryococcene. The notation denotes individual transformation events and the average of T_1_ lines assayed for that event. Specifically, the histobars represent botryococcene accumulation measured in three different leaves for each T_1_ plant with these values averaged together for all the T_1_ plants examined from that transformation event (±SE). Two or more different T_1_ plants were examined for each transformation event.

Lines that exhibited botryococcene accumulation were assayed again at 5 months old (Figure 7). Interestingly, botryococcene accumulation in lines engineered with only the BS gene were equal, if not greater than those engineered with both the BS+FPS genes. However, while botryococcene accumulation was easily observable, it did not approach the highest levels seen in *B. distachyon*.

## Discussion

These experiments have demonstrated the feasibility of producing a non‐native triterpene hydrocarbon (botryococcene) in the cytosol of the model monocot, *B. distachyon*. This is the first reported effort to engineer triterpene metabolism in this species as well as the first attempt to direct metabolism to specific subcellular compartments in a monocot. Unlike previous studies using the dicot, *Nicotiana tabacum* (Jiang *et al*., 2016), we were able to obtain higher titres of botryococcene by targeting both BS and FPS enzymes to the cytosol rather than to the plastid compartment (Figure 2; see Table 1 for results summary). Note that the apparent lower levels of botryococcene in T_1_ lines of Figure 2b is probably due in part to the younger age of plants examined there, versus the older age of plants as they arose out of tissue culture in Figure 2a. In an attempt to keep our interspecies‐specific comparisons as fair as possible, we have utilized highly expressed promoter complexes—like those highly expressed promoters used in the tobacco studies (Jiang *et al*., 2016; Wu *et al*., 2012)—well‐characterized for monocot engineering. While we did not observe increased transcript levels of putative genes involved in rate‐limiting steps of the MVA pathway (such as *HMGR*), it is possible that post‐translational modifications could compensate for the IPP and DMAPP shunted into specialized FPP and botryococcene biosynthesis. It would be interesting to compare enzyme activity levels of the MVA pathway steps in the high accumulating botryococcene lines to those in the wild type. Our observations with *B. distachyon* are also somewhat contrary to those of Masferrer *et al*. (2002) who noted that overexpression of an endogenous FPS in *Arabidopsis* resulted in a senescence‐like phenotype which was recoverable upon supplementation with exogenous mevalonate. It was later reported that a developmental decline in HMGR activity was correlated with this cell‐death phenotype, indicative of an inability of the MVA pathway to be up‐regulated in response to the elevated FPS activity and the resulting demand on intermediates like IPP and DMAPP (Manzano *et al*., 2004). Suggestive of differences in metabolic regulation between monocots and dicots, this phenotype was not observed here with overexpression of a heterologous FPS in *B. distachyon*.

**Table 1:** Summary of botryococcene accumulation and phenotypes observed in the two species examined with various engineering strategies

	Construct	Subcellular target	Highest botryococcene production (μg/g FW) ± SE	Phenotype
Brachypodium	BS	Cytosol	128 ± 9	WT
	BS + FPS	Cytosol	1177 ± 551	WT
	tpBS	Plastid	51 ± 8	WT
	tpBS + tpFPS	Plastid	112 ± 24	Chlorotic, stunted, premature senescence
Sorghum	BS	Cytosol	26 ± 10	WT
	BS + FPS	Cytosol	12 ± 2	WT
	tpBS + tpFPS	Plastid	ND	Recovered WT‐like plants, but no botryococcene

Because higher titres of botryococcene were observed in the BS + FPS lines compared to BS only lines (Figure 2), and because BS enzyme activity was not substantially different between these two engineered lines (Figure S3A), this suggests that FPP is normally limiting and unavailable for specialized metabolism as reflected in the case of the BS‐only expression lines. Overexpression of *BS + FPS*, must have been increased the flux of carbon to IPP and DMAPP in these transgenic lines in order to account for the increased levels of botryococcene accumulating, which could accumulate in comparable amounts to total phytosterols (compare Figures 2, 3). Moreover, because the phytosterols levels were unchanged in the BS+FPS lines relative to the BS‐only and wild type control, this indicates that there must be some regulatory mechanism controlling flux of the extra IPP and DMAPP to phytosterol biosynthesis in the BS+FPS lines as well.

While we did see botryococcene accumulation in *S. bicolor* plants engineered with cytosolic‐targeted BS and BS + FPS, we did not see an apparent effect from FPS overexpression in the lines examined (Figure 7). This could be due to only examining a limited number of transgenic lines, and thus the observed accumulation represents inherent variations in gene expression/enzyme activities and not be truly reflective of the full redirection capability of the MVA pathway in this species. It would be worthwhile to examine more independent lines of *S. bicolor*, comparing BS and BS + FPS engineering strategies. It should be noted, that the *S. bicolor* plants examined here were grown in the greenhouse under short, autumn days, and it is possible that optimizing the environmental inputs for this species could lead to increased botryococcene production (Jiang *et al*., 2017).

Perhaps the most striking observation from this study is the sensitivity of isoprenoid metabolism within the *B. distachyon* plastid. Targeting a heterologous FPS to the plastid induced a chlorotic phenotype that correlated with an inability to thrive on soil, so much so that it limited proliferation of leaf growth and seed development. While botryococcene accumulation was observed when both BS and FPS were targeted to the plastid, the inability of the plastid‐targeted FPS or plastid‐targeted BS + FPS plants to grow suggests it is the diversion of carbon within the plastid compartment that is important, rather than simply an effect of FPP accumulation. The inability to observe any transgenic *S. bicolor* plastid‐targeted BS + FPS lines supports the notion that this observation could be due to monocot‐specific sensitivities in plastid isoprenoid metabolism. Several possible explanations for the observed abnormal phenotype in these lines include: (i) a large redirection of carbon (glyceradehyde‐3‐phosphate and/or pyruvate) to the MEP pathway to compensate for the extra carbon sink directed to FPP biosynthesis could be limiting the biosynthesis of other more essential metabolites (like sugars and growth regulators); (ii) IPP/DMAPP being redirected to FPP rather than GGPP could limit the biosynthesis of carotenoids and chlorophylls essential for photosynthesis and may, in part, explain the chlorotic phenotype of the leaves; and (iii) the integrity of the plastid membrane could be especially sensitive to the presence of farnesol and/or botryococcene. In regard to latter possibility, while high levels of farnesol are known to inhibit plant growth through a detergent‐like activity on membranes (Hemmerlin and Bach, 2000; Hemmerlin *et al*., 2006), the fact that the plastid‐targeted plant lines could persist when supplied with an exogenous carbon source leads us to think that the abnormal plant phenotype is not due to a toxic build‐up of farnesol per se.

Why the cytosolic‐targeted approach to accumulate heterologous triterpene was so successful here and not in *N. tabacum* (and several other dicot species) is an intriguing question. It suggests that the *B. distachyon* MVA pathway is more malleable when metabolic sinks are introduced, and that control of carbon through the pathway may not be as tightly regulated as that in tobacco (Jiang *et al*., 2016; Wu *et al*., 2012). We reason that plants which produce prodigious amounts of specialized compounds from MVA‐derived IPP/DMAPP (like *N. tabacum*) most likely have tight feedback control over the output of the pathway when only a sink or ‘pull’ is introduced. In support of this, when a heterologous form of the rate‐limiting enzyme, HMGR, is expressed in tobacco it can drive accumulation of downstream, MVA‐pathway derived products (e.g. sterols or terpenes from heterologously‐expressed terpene synthases) (Chappell *et al*., 1995; Harker *et al*., 2003; Wu *et al*., 2006). Similar results have been observed in other species such as *Taraxacum brevicorniculatum* (Pütter *et al*., 2017) and *N. benthamiana* (Reed *et al*., 2017).

A novel compound beyond botryococcene was observed in the cytosolic‐engineered lines (peak 2, Figure 5a) which we interpret to be an epoxidated form of botryococcene, catalyzed by the endogenous SQE enzyme. Indeed, co‐expression of *BS* in combination with the putative *BdSQE1* in a yeast system yielded the same compound (Figure 6b). The ability of SQE enzymes to act on substrates with near terminal double bonds resembling squalene was reported some time ago (Van Tamelen and Heys, 1975). Upon examination of the mass spectrum for the oxidobotryococcene, mass ions were noted that could be indicative of epoxidation on either terminal bond and thus creating asymmetric isomers. This is perhaps not too surprising because Corey and Russey previously reported that 10,11‐dihydrosqualene could be mono‐ or di‐epoxidated at the terminal double bonds (Corey and Russey, 1966). The formation of dioxidosqualene has also been documented regardless of the kingdom of the SQE (Bai *et al*., 1992; Rasbery *et al*., 2007) and the formation of internally epoxidated squalenes have been observed in nature (De Napoli *et al*., 1982; Katayama and Marumo, 1976). Thus, the inherent promiscuity of SQE enzymes means it is not surprising to find an oxidobotryococcene present, especially when the BdSQE enzyme becomes saturated with high levels of botryococcene as in the case of the high accumulating *B. distachyon* lines.

This is the first report of an oxidobotryococcene generated in planta, which begged two additional questions. First, is oxidobotryococcene formed within *B. braunii* race B, the native algae where botryococcene is found naturally? We examined GC‐MS selected ion chromatograms (*m/z *=* *426) of hexane extracts from *B. braunii* and did observe the presence of small amounts of a constituent that could be the epoxide form of botryococcene. However, due to miniscule amounts of this signal and the large amounts of other compounds masking the MS signals, no definitive conclusion about epoxide forms of botryococcene in the native algal species can be made at this time. The second question pertains to the more intriguing possibility of botryococcene epoxide being utilized by OSCs for the generation of novel triterpene structures. Unfortunately, we did not find evidence for the generation of botryococcene‐derived cyclic triterpenes in either *B. distachyon* or in yeast. This does not mean it is not possible, only that the current conditions did not reveal any evidence for such a biochemical transformation. Hence, building novel triterpene scaffolds using combinations of unusual triterpene synthases like botryococcene synthase, SQE and OSCs remains a major conceptual and practical challenge.

We have demonstrated the ability to successfully produce the non‐native triterpene botryococcene in the cytosol of *B. distachyon* with amounts accumulating in mature plants upwards of 1 mg/g FW and in excess of the major phytosterol contents and without detrimental effects on plant development. In contrast, manipulation of isoprenoid metabolism in plastids of *B. distachyon* appears to be much less amenable to perturbations when compared to work done in tobacco (Jiang *et al*., 2016; Wu *et al*., 2006, 2012). While the cytosolic engineering was successful in *S. bicolor*, we have yet to observe the high levels seen with the model species. This could simply be a result of the limited number of transgenic lines assayed so far, and partially the growth parameters experienced by the *S. bicolor* plants in this experiment. Despite these results, one should be cautious in extrapolating our observations to fit all monocots. Indeed, variables like growth parameters, promoter choices, construct designs, and most critically, species examined all play important roles in dictating the phenomena observed. Potential future experiments examining species more closely related to *Brachypodium,* e.g. *Lolium* species (Bouchenak‐Khelladi *et al*., 2008), might elucidate the extent of evolutionary distance this capacity for metabolic redirection covers. Regardless, these results offer an important base to build upon, such as examining if the triterpene titres of the cytosolic engineered plants can be increased by introducing lipid‐droplet forming proteins, such as SEIPIN1 (Cai *et al*., 2015), or stacking a truncated HMGR and other upstream MVA pathway genes into the existing lines to increase flux into the MVA‐derived botryococcene (Chappell *et al*., 1995; Muñoz‐Bertomeu *et al*., 2007; Pütter *et al*., 2017; Reed *et al*., 2017; Wu *et al*., 2006).

While we have reported relatively high (~1 mg/g FW; 0.1% biomass) levels (to this point) of botryococcene in *B. distachyon* leaves, this titre is not substantial enough for commercialization. Production in *B. distachyon* has illustrated proof‐of‐concept for producing triterpenes in monocot/grass leaves, which based on the work done in *S. bicolor* could translate to crop species. A previous techno‐economic analysis of triterpene‐producing tobacco for biofuel use indicated a necessary threshold of productivity at 1% biomass with production increasing 10% every year to meet commodity threshold needs. As previously stated, using *B. braunii* as a production platform is not desirable because of its growth parameters/requirements. Thus, developing plant production platforms for these valuable compounds that grow robustly and utilize existing agricultural infrastructure still remains a considerable challenge (see companion manuscript, Kempinski and Chappell, Engineering triterpene metabolism in the oilseed of *Arabidopsis thaliana*).

## Experimental procedures

### Plant growth and transformation

Plants were grown in ProMix BX (Premier Tech) under 20 h light/4 h dark cycles for rapid flowering or 16 h light/8 h dark cycles for vegetative tissue bulking, using Sylvania OCTRON 6500K bulbs (Sylvania), with an approximate light intensity of 60 μmol/m^2^/s. Plants were watered as soil became dry and were fertilized every other watering with 20‐20‐20 general purpose fertilizer (The Scotts Company) diluted to 300 ppm N. Immature embryogenic plant callus tissue was transformed as described by Alves *et al*. (2009), with slight modifications: transformed calli were selected in the dark for three weeks (40 μg/mL hygromycin B) before moving to regeneration media under 16 h light conditions. Transformed calli on regeneration media were subcultured approximately every two to four weeks to fresh regeneration media for approximately 3 months or until plantlets appeared. Emerging plantlets were then moved to germination media (40 μg/mL hygromycin B). Robustly growing plantlets were then moved to soil.

*S. bicolor* transformation was done was previously described (Howe *et al*., 2006; Sato *et al*., 2004) at the University of Nebraska's Center for Plant Science Innovation. Regenerated T_0_ plants were allowed to set seed and the T_1_ seed was sent to the University of Kentucky in regulation with all necessary USDA‐APHIS interstate travel permits. The T_1_ seed was germinated on 1× MS media (Murashige and Skoog, 1962) (PhytoTechnology Laboratories) with 1% agar and G418 (10 μg/mL) under 16 h light/8 h dark as above. Plants were moved to a greenhouse at the University of Kentucky after three weeks, transplanted into ProMix BX, and allowed to grow under natural light in the fall of 2017. Plants were supplemented with fertilizer (as above) and ~0.1 mm Fe‐EDTA as needed.

### Vector construction

Plant transformation vectors were generated using the *pCXUN* vector backbone (Chen *et al*., 2009) using standard molecular methodologies. See Methods S1 for details of vector construction.

### Genomic DNA extraction and PCR analyses

All genomic DNA extractions were performed using a modified CTAB method similar to Porebski *et al*. (1997). All genotyping PCRs conducted on genomic DNA were done with Ex Taq polymerase (Takara) and with indicated primers. See Methods S2 for RNA extraction and RT‐PCR procedures.

### Cloning and yeast expression of *BdSQE1*

The putative *BdSQE1* (*BRADI1G69170*) gene was amplified from wild type plant cDNA using PrimeStar polymerase in a two‐step reaction with primers P42 + P43 according to the protocol provided by Takara. The purified product (Qiagen) was used as the template in another PCR to add a 5′ EcoRI site and a Kozak sequence (5′ AAAACA 3′), as well as a 3′ SpeI site for cloning into *pXHIS* (Zhuang and Chappell, 2015) with primers P44 +  P45. The cloned coding sequence was verified by DNA sequencing. All primers used in this study can be found in Table S1.

### Metabolite analyses

Botryococcene was extracted by harvesting plant tissue in a pre‐weighed 4 mL glass vial and grinding in liquid nitrogen. The tissue was allowed to warm to room temperature then weighed to obtain the fresh weight. A 1:1:1 mixture of hexane:acetone:water (1 mL each) was added to the sample (plus 5 μL of a 1 μg/μL hexadecane internal standard) and allowed to shake at room temperature for 20 min. The phases were separated by centrifugation at full speed in a clinical centrifuge, and the upper hexane phase removed and saved. Two more extractions with 1 mL hexane were performed and combined with the initial extract. The hexane extraction was dried under nitrogen gas, resuspended with iso‐octane, and transferred to a GC vial. Samples were analysed on either an Agilent 7890 GC (HP‐5MS column, 30 m x 0.25 mm, 0.25 μm film, 250 °C inlet temperature; oven temperature was 150 °C for 1:00 min, then 10 °C/min to 280 °C, then 5 °C/min to 310 °C and a final hold for 1:00 min; 0.9 mL/min He flow rate) connected to a 5975C Agilent mass spectrometer (run in positive ionization mode, 70 eV, scanning 50‐500 amu) or an Agilent 7890 GC (HP‐5 column, 30 m × 0.32 mm, 0.25 μm film; 60 °C for 1:00 min, then 30 °C/min to 230 °C, then 2 °C/min to 280 °C; 5.75 mL/min He flow rate) equipped with a flame ionization detector. Botryococcene in experimental samples was verified by comparing to an authentic botryococcene standard. Botryococcene accumulation was calculated based on an external standard curve run in tandem with the experimental samples.

Sterol levels were analysed by grinding plant tissue in liquid nitrogen, followed by saponification and sterol/tocopherol extraction as described by Du and Ahn (2002). The hexane extraction was dried to completion under nitrogen gas and samples were derivatized using a 1:1 mixture of pyridine and MSTFA + 1% TMCS (Thermo‐Fisher) at 50 °C for 1 h. Samples were analysed by GC‐MS (as described above) except the oven temperature was 200 °C initially, 10 °C/min to 270 °C, then 3 °C/min to 320 °C and held at 320 °C for 10 min. Major sterols were compared to known standards that had been derivatized identically as the experimental samples.

### Botryococcene purification and NMR analysis

T_1_ transgenic tissue from BS + FPS expressing *B. distachyon* lines was extracted as above, and 4 mg of botryococcene was purified and analysed by NMR. See Methods S3 for purification and NMR procedure details.

### Enzyme assays

BS enzyme assays were conducted by grinding harvested tissue in liquid nitrogen, then adding 5 μL/mg homogenization buffer (100 mm K_2_PO_4_ pH 7.0, 4 mm MgCl_2_, 250 mm sucrose, 5 mm β‐mercaptoethanol, 1× plant protease inhibitor cocktail VI [Bioworld]) and further homogenized. The homogenate was centrifuged at 4 °C at 2,000 × g for 5 min and 5 μL of this supernatant was used in a 50 μL reactions. The reaction conditions were modified from Niehaus *et al*. (2011), see Methods S4 for specific details.

## Conflicts of interest

Intellectual property has been secured by the University of Kentucky for some of this work and CK and JC are actively pursuing commercialization of this technology.

## Author contributions

J.C. and C.K. conceived the research plan and wrote the article; C.K. conducted the experiments; G.Z. conducted and analysed NMR data; and Z.J. provided conceptual/technical assistance and aided in data interpretation. S.J.S. and Z.G. conducted the sorghum transformation under the supervision of T.E.C.

## Supporting information


**Table S1** Primers used in this study.
**Figure S1** Developmental accumulation of botryococcene in cytosolic‐engineered lines. A) Botryococcene accumulation as a function leaf development as indicated by leaf length for a single T_1_ transgenic *B. distachyon* line (14‐1‐22) grown for ~3.5 months. B) Botryococcene accumulation in mature leaves of ~8‐month‐old T_0_ plants (compare overall levels to younger plants in Figure 2a). The average botryococcene levels in 3 independent samples of 2‐3 leaves were determined per indicated sample ± standard error.
**Figure S2** H^1^‐NMR spectrum of purified (4 mg, >98%) botryococcene from pooled BS + FPS T_1_ plant lines. Spectrum matches previous reports of published botryococcene H^1^‐NMR (Metzger *et al*., 1985).
**Figure S3** BS enzyme activity in transgenic lines targeting the BS activity to the cytoplasm or plastid compartments. A) BS specific activity in leaf lysates of T_0_ transgenic lines targeting BS (18‐1 and 18‐2) and BS + FPS (14‐4 and 14‐7) to the cytoplasm in comparison to non‐specific background activity in control (wild type) plants. B) BS specific activity in leaf lysates of transgenic T_0_ lines engineered for cytosol‐targeted BS + FPS (14‐4 and 14‐7) in comparison to a plastid‐targeted BS + FPS lines (16‐4). Also for comparison, the BS activity in bacterial lysates induced for expression of heterologous BS activity was included. Assays were run in triplicates and the averages plotted ± standard error. Different aged leaves were used for the activity measurements in graphs A and B.
**Figure S4** Normalized transcript levels of indicated endogenous *B. distachyon* genes in three botryococcene‐accumulating T_2_ plants (each from an independent transformation events) engineered with BS or BS + FPS and in WT. Histobars represent the average fold‐change of the gene of interest normalized to *UBI4* mRNA (2^−ΔCT^; ±standard deviation; *n* = three biological replicates). Significant differences in transcript levels compared to WT are indicated with * (α = 0.05, Student's *t*‐test).
**Figure S5** Hypothetical structures of oxidobotryococcenes. A) Predicted structure of 2,3‐oxidobotryococcene and putative mass fragments formed after electron ionization. B) Predicted structure of 20,21‐oxidobotryococcene and putative mass fragments formed after electron ionization. Mass fragments that would be indicative of either structure are boxed in yellow.
**Figure S6** Mass spectra of A) putative oxidobotryococcene from BSm + BdSQE1 transformed yeast and B) putative oxidobotryococcene from BdT0‐14‐7 (BS + FPS) transgenic plant line. The apparent molecular parent ion of m/z = 426 is indicated in both.
**Methods S1** Vector construction.
**Methods S2** RNA extraction and RT‐PCR analyses.
**Methods S3** Botryococcene purification and NMR analysis.
**Methods S4** Enzyme assays.
