# Supplementary material for: Engineering linear, branched‐chain triterpene metabolism in monocots
Source: Plant Biotechnol J. 2018 Oct 16;17(2):373–85. doi: 10.1111/pbi.12983 (PMC6335073; doi:10.1111/pbi.12983)
Supplement: Supplementary file 1 — Table S1 Primers used in this study. Figure S1 Developmental accumulation of botryococcene in cytosolic‐engineered lines. A) Botryococcene accumulation as a function leaf development as indicated by leaf length for a single T1 transgenic B. distachyon line (14‐1‐22) grown for ~3.5 months. B) Botryococcene accumulation in mature leaves of ~8‐month‐old T0 plants (compare overall levels to younger plants in Figure 2a). The average botryococcene levels in 3 independent samples of 2‐3 leaves were determined per indicated sample ± standard error. Figure S2 H1‐NMR spectrum of purified (4 mg, >98%) botryococcene from pooled BS + FPS T1 plant lines. Spectrum matches previous reports of published botryococcene H1‐NMR (Metzger et al., 1985). Figure S3 BS enzyme activity in transgenic lines targeting the BS activity to the cytoplasm or plastid compartments. A) BS specific activity in leaf lysates of T0 transgenic lines targeting BS (18‐1 and 18‐2) and BS + FPS (14‐4 and 14‐7) to the cytoplasm in comparison to non‐specific background activity in control (wild type) plants. B) BS specific activity in leaf lysates of transgenic T0 lines engineered for cytosol‐targeted BS + FPS (14‐4 and 14‐7) in comparison to a plastid‐targeted BS + FPS lines (16‐4). Also for comparison, the BS activity in bacterial lysates induced for expression of heterologous BS activity was included. Assays were run in triplicates and the averages plotted ± standard error. Different aged leaves were used for the activity measurements in graphs A and B. Figure S4 Normalized transcript levels of indicated endogenous B. distachyon genes in three botryococcene‐accumulating T2 plants (each from an independent transformation events) engineered with BS or BS + FPS and in WT. Histobars represent the average fold‐change of the gene of interest normalized to UBI4 mRNA (2−ΔCT; ±standard deviation; n = three biological replicates). Significant differences in transcript levels compared to WT are indicated with * (α = 0.05, [file PBI-17-373-s001.docx]

Supporting information

Table S1. Primers used in this study.

| **Primer** | **Sequence (5' -> 3')** | **Comment** |
| --- | --- | --- |
| P1 | ATGCAGCCCCATCATCAT | aFPS_CDS_F |
| P2 | TCATTTCTGGCGTTTGTAGATC | aFPS_CDS_R |
| P3 | ATGACTATGCACAAGACCACGG | BbSSL1_F1_incorrect |
| P4 | TCAAGCACCCTTAGCTGAAACC | BbSSL3_R1 |
| P5 | GTCTTGCAGCACCCGCGTGAGATTATCCCTC | Removes XmaI site from BS |
| P6 | GAGGGATAATCTCACGCGGGTGCTGCAAGAC | Removes XmaI site from BS |
| P7 | CAGACCCAAAGCTACTGGACCGGGAGG | Removes HindIII site from BS |
| P8 | CCTCCCGGTCCAGTAGCTTTGGGTCTG | Removes HindIII site from BS |
| P9 | ATGACTATGCACCAAGACCACGG | BS_F |
| P10 | ATGGCGCCCACCGTGATGATGGCCTCGTCGGCCACC | Amplifying ZmRbcsTp CDS from gDNA |
| P11 | CTAGTCGCTGCCCGGGGGCTTGTAGGCGATGAAGCT | Amplifying ZmRbcs CDS from gDNA |
| P12 | GGATCCGGTGCATGCAGCCCCATCATCATCATAAAGAGGGG | BamHI-ZmRbcs-GgFPS_F1 |
| P13 | TCAAAGATCTTCTTCAGAAATCAACTTTTG | CAG-FPS-MYC-TGA_R2 |
| P14 | GGCGGAAGGATCCGGTGCATGCAGCC | ZRTp_mutation_del_F |
| P15 | GGCTGCATGCACCGGATCCTTCCGCC | ZRTp_mutation_del_R |
| P16 | ATGGCGCCCACCGTGATGATG | ZmRTp_F |
| P17 | TCATTTCTGGCGTTTGTAGATCTTC | GgFPS_3'end |
| P18 | GCACCGGATCCTTCCGCCGTTGCTGACGT | ZmRbcs_R1 |
| P19 | CAACGTCAGCAACGGCGGAAGGATCCGGTGCATGACTATG | ZRTp-5'addition-SSL1_F1 |
| P20 | TCAAGCACCCTTAGCTGAAACCTTTCCATTT | BbSSL3_R1r |
| P21 | GAAGCCAACTAAACAAGACCATAACCATGGTG | PvUbi2-prom_F |
| P22 | CTGCAAAAGAGAACCAGACAACAGGG | PvUbi2-prom_R |
| P23 | ATTTTCCAAGCTTGAAGCCAACTAAACAAGACCATAACC | HindIII-PvUbi2_F2 |
| P24 | ACCGGTCTGCAGCAAAAGAGAACCAGACAACAGGGT | PU2p5i-PstI-AgeI_R |
| P25 | ATTTTCCAAGCTTGATCTAGTAACATAGATGACACCGCGCGCG | HindIII-NOSt_R2 |
| P26 | ATACTAGTGAAGCCAACTAAACAAGACCATAA | AT-SpeI-PU2p F |
| P27 | ATACCGGTAAAACGACGGCCAGTGAATT | AT-AgeI-pC-NOSt R |
| P28 | TGACACCATCGACAACGTGA | BdUBI4 qRT F |
| P29 | GAGGGTGGACTCCTTCTGGA | BdUBI4 qRT R |
| P30 | GTTGGATTACCTCCAGGATGAC | BRADI3G40210 (HMGR1) qRT F |
| P31 | CGTTGATTACTGCCTCACAAAC | BRADI3G40210 (HMGR1) qRT R |
| P32 | GACCTTCACATCTCGGTTACTATG | BRADI4G33910 (HMGR2) qRT F |
| P33 | GATCCTGGAGATTCCCTGTTTG | BRADI4G33910 (HMGR2) qRT R |
| P34 | GACCCAAGTGCTGCGATAA | BRADI1G04878.1 (SQS) qRT F |
| P35 | GAGCCACCAGCATAAGTACAA | BRADI1G04878.1 (SQS) qRT R |
| P36 | GGGAATACAGGCCGAAGATTTA | BRADI2G25360 (IDI1) qRT F |
| P37 | CGAACCATGAACAGAAGGTAGT | BRADI2G25360 (IDI1) qRT R |
| P38 | GTACCGTGAATCCGAGCTTATC | BRADI1G25350 (IDI2) qRT F |
| P39 | CGTCAACTGGCACATCCTT | BRADI1G25350 (IDI2) qRT R |
| P40 | GGATGGCCTTTGGGATGTAT | BRADI5G02170.1 (AACT) qRT F |
| P41 | GCATAAGCGTCCTGGTCTT | BRADI5G02170.1 (AACT) qRT R |
| P42 | ATGGCTGCGGCCGCTGGCTTCGGGC | BdSQE1_CDS_F |
| P43 | TCAGAACTCCGCTTCAGGAGGAGCC | BdSQE1_CDS_R |
| P44 | ATAGAATTCAAAACAATGGCTGCGGCCGCTGGCTTCGGGC | BdSQE1 F (5' EcoRI/5' Kozak) |
| P45 | ATAACTAGTTCAGAACTCCGCTTCAGGAGGAGCC | BdSQE1 R, 3' SpeI |


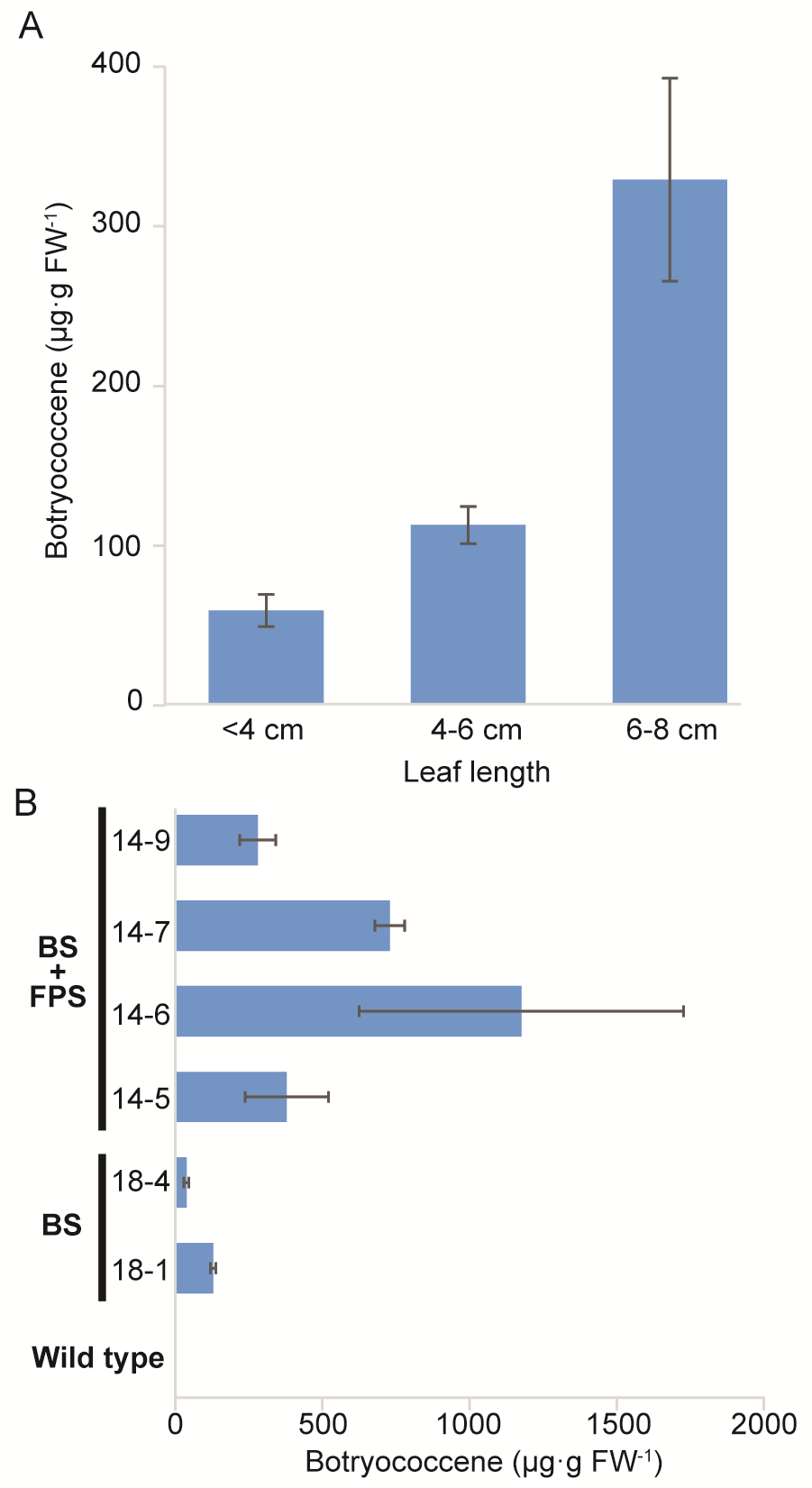


Figure S1. Developmental accumulation of botryococcene in cytosolic-engineered lines. A) Botryococcene accumulation as a function leaf development as indicated by leaf length for a single T_1_ transgenic *B. distachyon* line (14-1-22) grown for ~3.5 months. B) Botryococcene accumulation in mature leaves of ~8-month-old T_0_ plants (compare overall levels to younger plants in Figure 2A). The average botryococcene levels in 3 independent samples of 2-3 leaves were determined per indicated sample ± standard error.


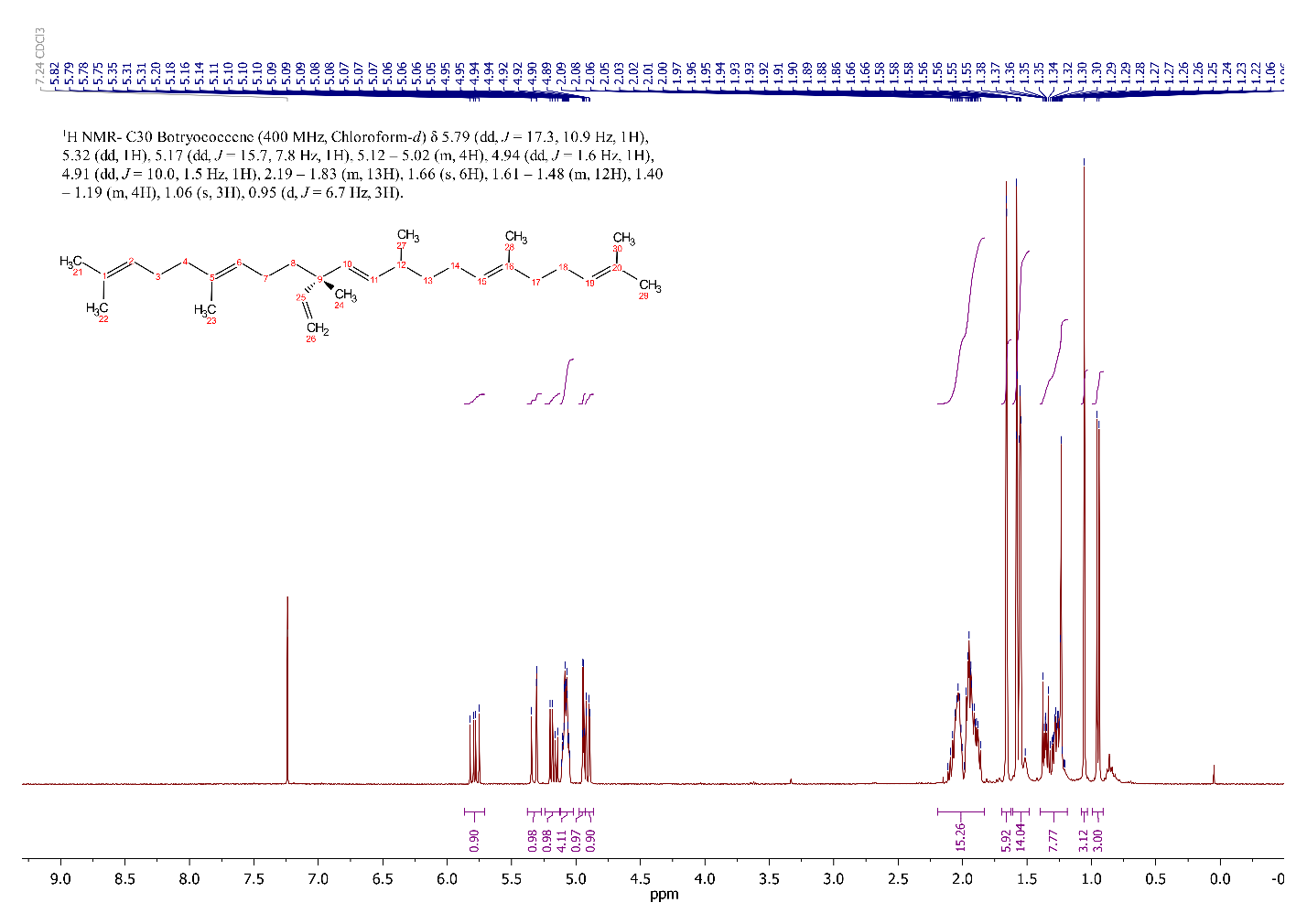


Figure S2. H^1^-NMR spectrum of purified (4 mg, >98%) botryococcene from pooled BS + FPS T_1_ plant lines. Spectrum matches previous reports of published botryococcene H^1^-NMR (Metzger et al., 1985).

**Figure S3.** BS enzyme activity in transgenic lines targeting the BS activity to the cytoplasm or plastid compartments. A) BS specific activity in leaf lysates of T0 transgenic lines targeting BS (18-1 and 18-2) and BS + FPS (14-4 and 14-7) to the cytoplasm in comparison to non-specific background activity in control (wild type) plants. B) BS specific activity in leaf lysates of transgenic T0 lines engineered for cytosol-targeted BS + FPS (14-4 and 14-7) in comparison to a plastid-targeted BS + FPS lines (16-4). Also for comparison, the BS activity in bacterial lysates induced for expression of heterologous BS activity was included. Assays were run in triplicates and the averages plotted ± standard error. Different aged leaves were used for the activity measurements in graphs A and B.


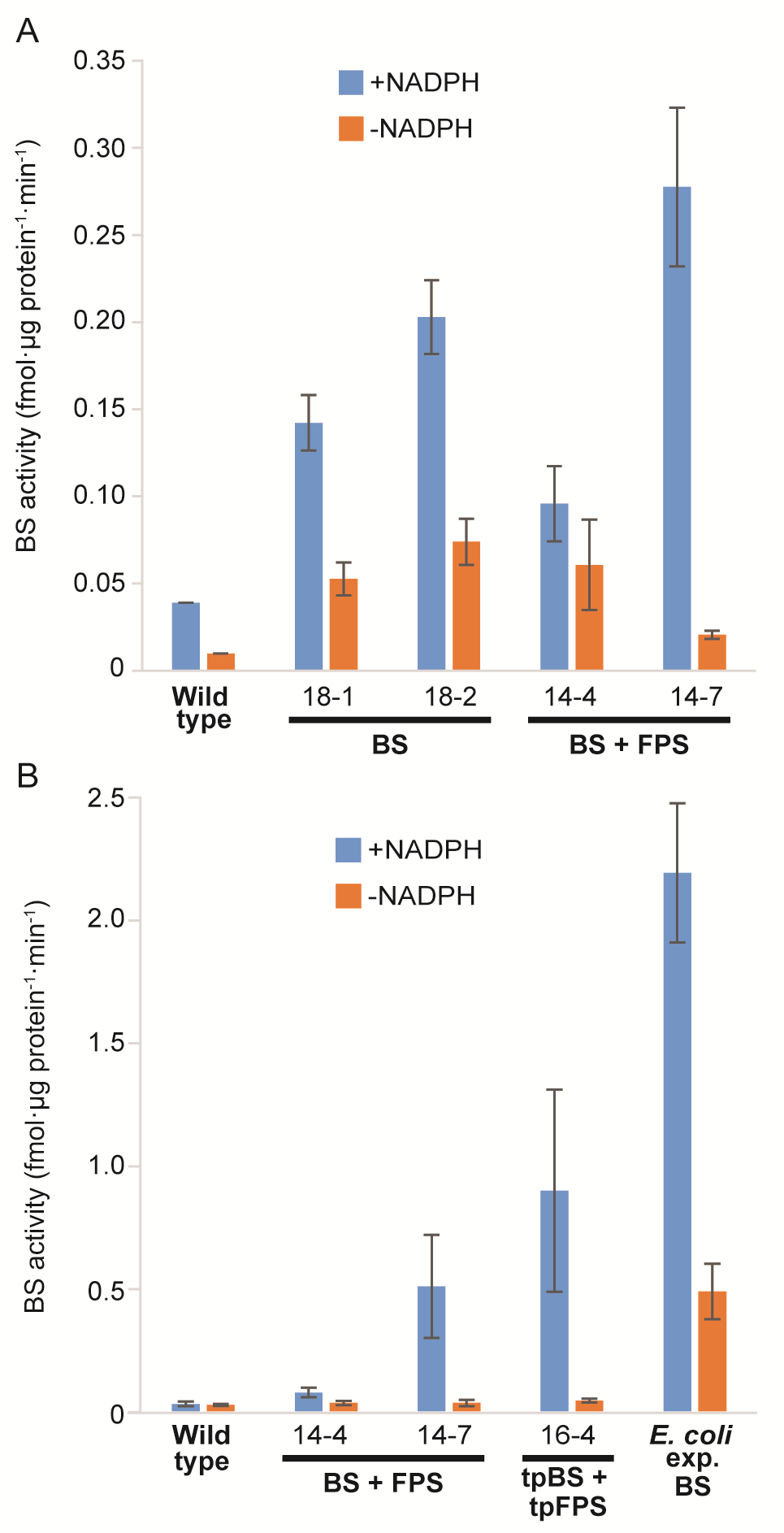


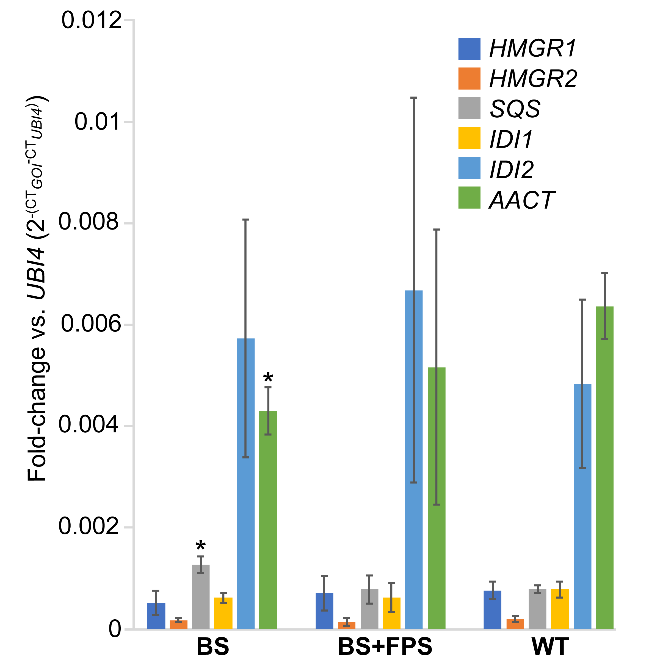


Figure S4. Normalized transcript levels of indicated endogenous *B. distachyon* genes in three botryococcene-accumulating T_2_ plants (each from an independent transformation events) engineered with BS or BS + FPS and in WT. Histobars represent the average fold-change of the gene of interest normalized to *UBI4* mRNA (2^-ΔCT^; ±standard deviation; n = three biological replicates). Significant differences in transcript levels compared to WT are indicated with * (α=0.05, Student’s *t*-test).


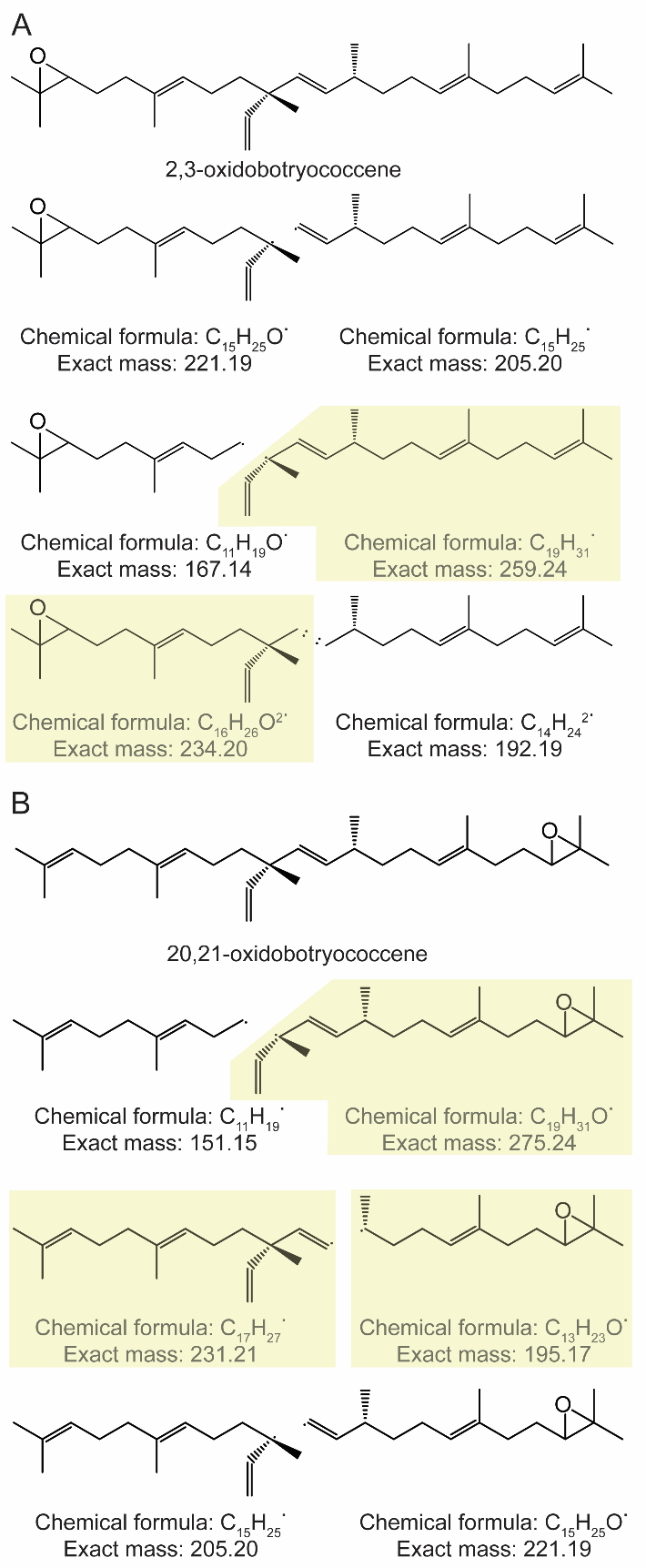
Figure S5. Hypothetical structures of oxidobotryococcenes. A) Predicted structure of 2,3-oxidobotryococcene and putative mass fragments formed after electron ionization. B) Predicted structure of 20,21-oxidobotryococcene and putative mass fragments formed after electron ionization. Mass fragments that would be indicative of either structure are boxed in yellow.


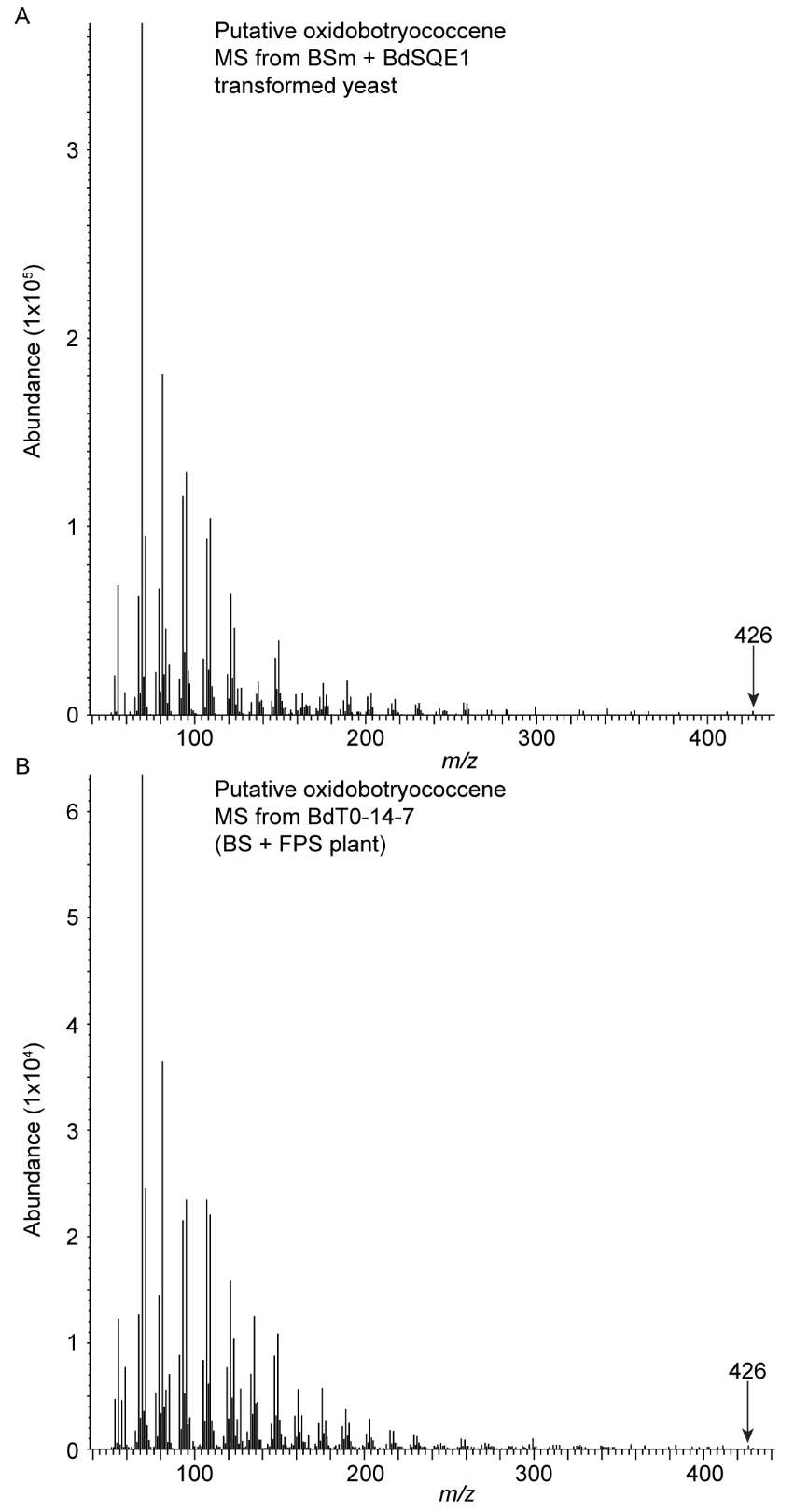
**Figure S6**. Mass spectra of A) putative oxidobotryococcene from BSm + BdSQE1 transformed yeast and B) putative oxidobotryococcene from BdT0-14-7 (BS + FPS) transgenic plant line. The apparent molecular parent ion of *m/z* = 426 is indicated in both.

**Supporting Methods**

**Methods S1 Vector construction**

The coding sequence for the *Gallus gallus FPS* (Tarshis et al., 1994; GenBank ID: 425061) was amplified from a plasmid harboring the plastid-targeted *FPS* used by Wu et al., (2012) using primers P1 + P2 (all primers can be found in Supplemental Table 1) with Primestar polymerase as recommended by the manufacturer (Takara) and purified using Qiagen PCR purification kit (Qiagen). The purified amplicon was ligated in the XcmI-digested pCXUN vector (the *ccdB* gene was previously removed) after 3’-tailing adenines using Ex Taq polymerase (Takara) in a modified PCR with 2.5 mM dATP and incubated at 70^o^C for 25 minutes. All ligations were done using T4 DNA ligase per the manufacturer’s protocol (Promega). Ligations were transformed into *Escherichia coli* strain DH5α, and putative positive clones with desired insert orientation were checked by colony PCR. This cloning effort created *pC-ZU1p5i::FPS*. The coding sequence for the *SSL1-3* gene fusion (designated here as *BS*, see Niehaus et al., [2011]) was amplified using primers P3 + P4 and cloned into the *pGEM* vector via 3’-tailing adenines as described above to create *pG-BS*. The XmaI and HindIII sites in *BS* were subsequently removed by making synonymous mutations using successive rounds with the Quikchange Lightening Mutagenesis kit (Agilent) using primers P5 + P6 to remove XmaI, and P7 + P8 to remove HindIII. This altered version of *BS* was amplified using P9 + P4 primers (P9 corrected an error introduced by P3) and cloned into *pCXUN* yield *pC-ZU1p5i::BS*.

To add the *Zea mays* RuBisCO small subunit transit gene sequence encoding for its corresponding plastid targeting sequence peptide (tp; Lebrun et al., 1987; Wostrikoff et al., 2012) onto the BS and FPS genes, the entire RuBisCO small subunit gene (GenBank ID: 542212) was amplified from *Z. mays* genomic DNA (primers P10 + P11) and cloned, from which the *tp* encoding sequence was amplified (primers P12 + P13) and inserted in-frame at the 5’ terminus of the *FPS* gene to generate *pG-BamHI-tpFPS-C-FLAG*. Both *pG-tp* and *pG-BamHI-tpFPS-C-FLAG* were digested with BamHI and NdeI (NEB), generating *pG-tp-BamHI-NdeI* and *BamHI-tpFPS-C-FLAG-NdeI*, respectively. Gel purified *BamHI-tpFPS-C-FLAG-NdeI* segment was ligated into the *pG-tp-BamHI-NdeI* vector creating *pG-tpFPS-C-FLAG*. After sequencing, this vector was observed to have a single nucleotide mutation resulting in a frameshift which was corrected by amplifying the *pG-tpFPS-C-FLAG* vector with primers P14 + P15 with 3% DMSO in the PCR. The incorrect template vector was removed from the reaction by adding DpnI (NEB) directly to the PCR, incubating at 37^o^C for 1 hour, and transforming the entire reaction into DH5α. The untagged *tpFPS* gene was amplified using primers P16 + P17 and ligated into XcmI-digested *pCXUN* as described above, creating *pC-ZU1p5i::tpFPS*.

To add the *tp* to the 5’ end of *BS*, a splicing by overlap extension (Horton et al., 1989) approach was used. The *tp* was amplified with primers P16 + P18 and *BS* with a 5’ *tp* overhang was amplified with P19 + P20. The two gel purified reaction products were combined in a 2:1 (*tp:tp-overhang-BS*) ratio and the full *tpBS* was amplified using primers P16 + P20, and the desired product was gel purified. The *tpBS* gene was cloned into *pCXUN* as described above and checked for the desired orientation by PCR — this created *pC-ZU1p5i::tpBS*.

The *Zea mays Ubiquitin1* promoter, 5’ UTR, and intron 1 (*ZU1p5i*) was removed from *pC-ZU1p5i::BS* and *pC-ZU1p5i::tpBS* by HindIII and XmaI digestion (NEB) and replaced with the *Panicum virgatum Ubiquitin2* promoter consisting of the 5’ UTR, and intron 1 sequence (*PU2p5i*; Mann et al., 2011) amplified from *P. virgatum* (cultivar: Alamo) using primers P21 + P22. A HindIII restriction site was added to the 5’ end of *PU2p5i* and a PstI restriction site was added to the 3’ end of *PU2p5i* to create a 3’ splice site necessary for intron excision, as well as inclusion of an AgeI site 3’ to the PstI site. These were added by amplifying *PU2p5i* with primers P23 + P24, and the corresponding product was purified, 3’-tailed with adenines and ligated into the *pGEM* vector to yield *pG-HindIII-PU2p5i-PstI-AgeI*. The *HindIII-PU2p5i-PstI-AgeI* fragment was subsequently isolated by digestion with HindIII and AgeI and ligated into the HindIII/XmaI digested *BS* and *tpBS* vectors to generate *pC-PU2p5i::BS* and *pC-PU2p5i::tpBS*, respectively.

The double expression vectors were generated by digesting the *pC-ZU1p5i::FPS* and *pC-ZU1p5i::tpFPS* with HindIII, followed by treatment with calf intestine alkaline phosphatase (CIAP; Promega). The *PU2p5i::BS:NOSt and PU2p5i::tpBS:NOSt* fragments were amplified from their respective pC-vectors using the primers, P23 + P25, digested with HindIII (NEB) and ligated into the HindIII/CIAP-digested vectors to create the *pC-PU2p5i::BS + ZU1p5i::FPS* vector and the *pC-PU2p5i::tpBS + ZU1p5i::tpFPS* vector. The cytosol-targeted dual expression vectors had the two gene cassettes placed in tandem in the same 5’ to 3’ direction, while the plastid-targeted vectors had the two cassettes oriented in opposite directions. The integrity of vectors verified by sequencing prior to plant transformation.

Constructs for transformation into *S. bicolor* utilized the pZP212 T-DNA vector backbone (Hajdukiewicz et al., 1994). Briefly, the *PU2p5i::BS*, *PU2p5i::BS* + *ZU1p5i::FPS*, and *PU2p5i::tpBS* + *ZU1p5i::tpFPS* expression cassettes were amplified from the *pCXUN* vectors described above using primers P26 +P27 (note: the *PU2p5i::tpBS + ZU1p5i::tpFPS* used here was in the tandem orientation, not the opposite as above). These fragments were digested with SpeI and AgeI before being ligated into pZP212 vector digested with XmaI and XbaI. The coding regions of the genes were confirmed by sequencing prior to plant transformation.

**Methods S2 RNA extraction and RT-PCR analyses**

Second and third leaves from 3-4 leaf culms of T_2_ lines 18-3-13-4, 18-5-5-5, 18-11-12-6, 14-6-5-9, 14-9-13-7, and 14-14-6-3 (as well as WT) were flash frozen in liquid nitrogen, powdered in liquid nitrogen and RNA extracted by homogenization in 1 mL of Tri-Reagent according to the manufacturer’s protocol (Molecular Research Center). RNA integrity was checked using agarose gel electrophoresis and concentration determined with a NanoDrop spectrophotometer (Thermo-Fisher). Following DNAseI treatment, 500 ng for the RNA samples was used to generate first-strand cDNA with random hexamer primers and SuperScriptIII (Invitrogen). The equivalent of approximately 5.95 ng of RNA was used as the template in PCR reactions to amplify specific target mRNAs. Primers for endogenous genes used in qRT-PCR are listed in Supplementary Table 1, P28-P41. All primers were designed for this study except those for *UBI4* (Hong et al. 2008). Reactions were set up using an Eppendorf 5070 liquid handler and ran on an Applied Biosystems 7900HT real-time PCR machine using SYBR green as a reporter. C_T_ values were calculated automatically by the 7900HT software. The putative endogenous *B. distachyon* isoprenoid genes examined were based on a BLAST search using the respective *Arabidopsis thaliana* homologs against the *B. distachyon* genome in the Gramene database (http://www.gramene.org). The specific genome locus for each gene examined is given in Supplemental Table 1.

**Methods S3 Botryococcene purification and NMR analysis**

Approximately 100 g of T1 transgenic (BS + FPS) B. distachyon tissues (pooled from different transgenic events which exhibited high botryococcene accumulation) was powdered in liquid nitrogen, then extracted with 3 L of 1:1:1 ratio of water:acetone:hexane and the hexane phase recovered with a separatory flask. The tissue residue was extracted with another 1 L of hexane and combined with the first extract. This process was repeated with another ~100 g of tissue and the total hexane extracts were combined and condensed to ~100 mL with a rotoevaporator. To remove precipitates, the extract was flashed through ~1 g silica columns in aliquots using nitrogen gas. The columns were flushed with hexane and the combined, filtered extract again condensed to ~15 mL with a rotoevaporator. This solution was added to a 5 g silica column (~10 mL column volume) and the column was eluted with ~120 mL of hexane. Twenty mL fractions were collected and each analyzed by GC-MS. Botryococcene was in the first two fractions, which were combined and condensed to ~2 mL. This sample was run on a preparative TLC plate (Silica G, glass baked, 1 mm thickness), developed with hexane. The botryococcene zone (RF = 0.48 – 0.57) was scraped from the plate and eluted with hexane. The final purity of the botryococcene was assessed using GC-MS to be greater than 95% pure and 4 mg was analyzed using a 1H-NMR (400 MHz, suspended in chloroform-d).

**Methods S4 Enzyme assays**

The BS enzyme assays consisted of 50 mM MOPs (pH 7.3), 20 mM MgCl_2_, 2.5 mM β-mercaptoethanol, 2 mM NADPH (omitted in negative control reactions), 5 μM [1-^3^H]-FPP (approximately 4 x 10^5^ DPM total), 2 mM imidazole, and 2 mM sodium orthovanadate. Plant lysate was added to prepared reactions (with or without NADPH) and allowed to proceed for 10 minutes at 37^o^C. Reactions were stopped by adding one volume 250 mM EDTA. Botryococcene was extracted with two volumes of *n*-hexane (containing 0.1 μg·μL^-1^ botryococcene). Fifty μL of the *n*-hexane extract was spotted on a silica G TLC plate and developed with *n*-hexane. The botryococcene zone was visualized with iodine vapor (verified by comparison to an external standard) then scraped and counted by scintillation spectrometry. Total protein in the crude lysate was quantified using the Bio-Rad protein reagent (Bio-Rad). BS expressed in *E. coli* was generated by using a pET28a construct from Bell et al. (2014). Briefly, BL21(DE3) cells were transformed with the pET28a-BS plasmid and independent colonies were used to inoculate 1.5 mL of LB + kanamycin (100 μg/mL), which were grown at 37^o^C, 220 rpm for ~23 hours (until OD_600_ was ~0.5). The entire 1.5 mL culture was used to inoculate a 50 mL LB + kanamycin (100 μg/mL) culture which was grown at 37^o^C, 220 rpm for ~2.8 hours (OD_600_ ~0.5-1.0). The cultures were split in half with one culture being induced with 1 mM IPTG. The cultures were grown at 23^o^C, 220 rpm for ~18 hours. After this, the cultures were divided into aliquots and the cells collected and frozen at -80^o^C until needed. For enzyme activity, cell pellets were resuspended in 1 mL of cold reaction buffer (50 mM MOPS [pH 7.3], 2.5 mM β-mercaptoethanol, and 20 mM MgCl_2_), sonicated six times, 20% power, 20 second bursts (Fisher Scientific model: FB505), and cooled on ice for 2 minutes between sonications. Lysates were spun at 10,000 x g for 10 minutes at 4^o^C and 5 μL of the supernatant used in the enzyme assays.

**Supplemental References**

**Bell SA, Niehaus TD, Nybo SE, Chappell J** (2014) Structure–function mapping of key determinants for hydrocarbon biosynthesis by squalene and squalene synthase-like enzymes from the green alga Botryococcus braunii race B. Biochemistry **53**: 7570–7581

**Hajdukiewicz P, Svab Z, Maliga P** (1994) The small, versatile pPZP family of Agrobacterium binary vectors for plant transformation. Plant Mol Biol **25**: 989–994

**Hong S-Y, Seo PJ, Yang M-S, Xiang F, Park C-M** (2008) Exploring valid reference genes for gene expression studies in Brachypodium distachyon by real-time PCR. BMC Plant Biol **8**: 112

**Horton RM, Hunt HD, Ho SN, Pullen JK, Pease LR** (1989) Engineering hybrid genes without the use of restriction enzymes: gene splicing by overlap extension. Gene **77**: 61–68

**Lebrun M, Waksman G, Freyssinet G** (1987) Nucleotide sequence of a gene encoding corn ribulose-1,5-bisphosphate carboxylase/oxygenase small subunit (rbcs). Nucleic Acids Res **15**: 4360

**Mann DGJ, King ZR, Liu W, Joyce BL, Percifield RJ, Hawkins JS, LaFayette PR, Artelt BJ, Burris JN, Mazarei M, et al** (2011) Switchgrass (Panicum virgatum L.) polyubiquitin gene (PvUbi1 and PvUbi2) promoters for use in plant transformation. BMC Biotechnol **11**: 74

**Metzger P, Casadevall E, Pouet MJ, Pouet Y** (1985) Structures of some botryococcenes: branched hydrocarbons from the b-race of the green alga Botryococcus braunii. Phytochemistry **24**: 2995–3002

**Niehaus TD, Okada S, Devarenne TP, Watt DS, Sviripa V, Chappell J** (2011) Identification of unique mechanisms for triterpene biosynthesis in Botryococcus braunii. Proc Natl Acad Sci **108**: 12260–12265

**Tarshis LC, Yan M, Poulter CD, Sacchettini JC** (1994) Crystal structure of recombinant farnesyl diphosphate synthase at 2.6-A resolution. Biochemistry **33**: 10871–10877

**Wostrikoff K, Clark a., Sato S, Clemente T, Stern D** (2012) Ectopic Expression of Rubisco Subunits in Maize Mesophyll Cells Does Not Overcome Barriers to Cell Type-Specific Accumulation. Plant Physiol **160**: 419–432
